# Predicting Lipid Eutectics Using Coarse-Grained Molecular Dynamics

**DOI:** 10.1021/acs.jpcb.3c06297

**Published:** 2023-11-17

**Authors:** Robert J. Cordina, Beccy Smith, Tell Tuttle

**Affiliations:** † Cadbury UK Ltd., P.O. Box 12, Bournville Lane, Birmingham B30 2LU, U.K.; ‡ Department of Pure and Applied Chemistry, University of Strathclyde, 295 Cathedral Street, Glasgow G1 1XL, U.K.

## Abstract

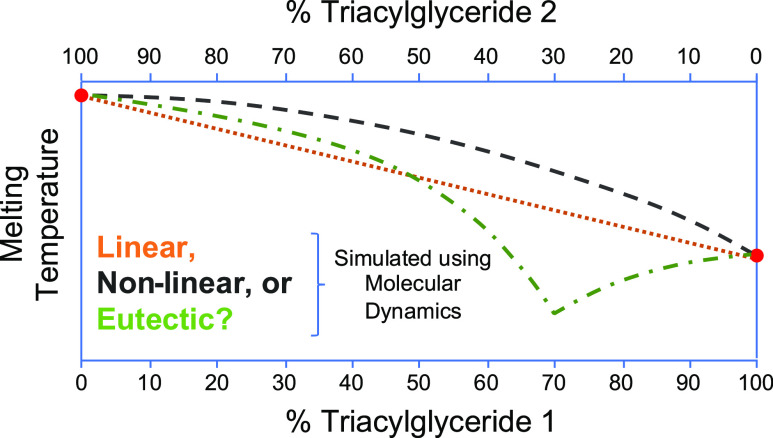

Binary triacylglyceride (TAG) systems are known to exhibit nonlinear/eutectic behavior, and predicting this is not straightforward unless the thermodynamic properties of the constituent TAGs are known. While this kind of behavior has been shown previously using molecular dynamics, this was carried out using an atomistic force field and fully saturated symmetric TAGs. In this study, we simulate the changing melting point of binary unsaturated asymmetric TAG systems using a coarse-grained force field. While the simulated melting temperatures of the various TAG ratios are generally found to be less than the empirical values, the nonlinear/eutectic behavior is reproduced very well for the three different binary TAG systems used. Hence, this opens up the possibility of being able to simulate the behavior of different, unknown TAG systems.

## Introduction

Composite materials, organic or inorganic in nature, are known to exhibit nonlinear behavior with a changing ratio of the components.^1−4^ That is, if two pure materials, be they elements such as lead and tin^3^ or ionic liquids,^4^ each with their own defined but different melting point, are mixed together, then the melting point of the mixture cannot be predicted by a simple weighted arithmetic mean. Such nonlinear behavior can extend to a eutectic point, i.e., the melting point of the mixture at a specific ratio of the components is lower than the melting point of either of the pure components. This melting point depression may be desired, such as in lead solder^3^; however, this may not always be the case.

**Figure 1:**
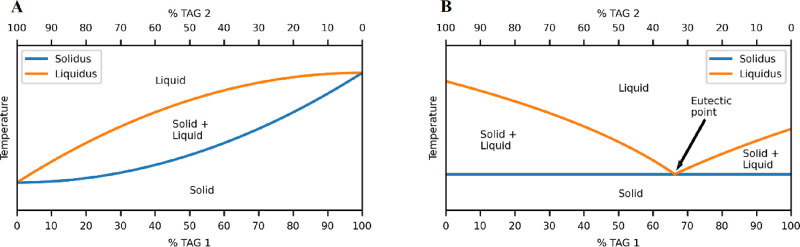
Examples of (A) nonlinear mixing behavior and (B) eutectic behavior of two TAGs with different pure melting points. The top line is the liquidus (point at which the mixture is fully melted), and the bottom line is the solidus (point at which the mixture starts to melt). Adapted from Machridachis-González et al.^12^ Copyright 2020, MDPI.

Similarly, fats, due to their composite triacylglyceride (TAG) composition, are known to not only melt over a temperature range, generally given as a solid fat content profile (a plot of solid fat content versus temperature), but this temperature range will change with changing TAG composition.^5−7^ Their behavior, however, is not easy to predict by simply knowing the composition of the mixture’s composition. Simplifying fat systems to a binary TAG system, the melting behavior is generally shown in a phase diagram (such as in Figure 1), where the solidus line is the point at which the mixture starts to melt, and the liquidus line is the point at which the mixture is fully melted out, with the melting temperature range changing with TAG ratio. Binary TAG mixtures can show both nonlinear and eutectic behavior, exemplified in Figure 1A,B, respectively. Such binary TAG mixture behavior has been tested empirically by various groups on different types of TAGs, including symmetric and asymmetric and saturated and unsaturated TAGs.^8−15^

The behavior exhibited by any given TAG combination, or at which TAG ratio the eutectic point will be found, however, is not immediately obvious to predict by a simple analysis of the TAG structures. This means that the melting point for a mixture of TAGs at a given ratio must be either determined empirically or, as in this study, simulated for. Mathematical models, such as the Hildebrand model,^16^ to predict the nonlinear behavior have been applied to binary TAG mixtures^10^; however, these still require knowledge of the melting temperature and enthalpy of fusion of the pure components, which may not always be available.

In a previous study, we have shown that the melting point of various TAGs can be predicted using the using the COarse-Grained Interchangeable Triglyceride-Optimized (COGITO)^17^ force field (FF).^18^ In this study, we have extended this and investigated the nonlinear behavior of binary TAG mixtures of 1-palmitoyl-2-oleoyl-3-stearoyl-*sn*-glycerol (*sn*-POSt), 1,3-dipalmitoyl-2-oleoyl-*sn*-glycerol (*sn*-POP), and 1,3-distearoyl-2-oleoyl-*sn*-glycerol (*sn*-StOSt) using the same FF. These are the three main TAGs found in cocoa butter, and their nonlinear behavior has been studied extensively^8,10−12,19^ given their importance in the confectionery industry, thus allowing for an easy comparison of the simulated results with empirical data.

## Computational Methods

### Molecular Dynamics (MD) Simulation Settings

All simulations have been carried out using GROMACS^20^ 2021.3 with the COGITO FF.^17^ All equilibrations were done using an NPT ensemble using a time step of 25 fs and a pressure of 1.01325 bar using a v-rescale thermostat and a Berendsen barostat. Anisotropic pressure coupling, with a compressibility of 1 × 10^–5^ bar^–1^ in the *x*, *y*, and *z* directions, was used. Temperature coupling was set at 1 ps, while pressure coupling was set at 10 ps. The cutoff scheme was set to Verlet, with the Coulomb and vdW cutoff distances set to 1.1 nm. The vdW modifier was set to potential shift, and the electrostatics (Coulomb type) was set to particle-mesh Ewald (PME) (PME order = 4). All systems were heated from 248 to 348 K over 200 ns, giving a linear heating rate of 0.5 K/ns (5 × 10^8^ K/s).

### Building Binary Mixtures

The coarse-grained representations of *sn*-POP, *sn*-POSt, and *sn*-StOSt were built by mapping the XRD crystalline structures^21^ as per Cordina et al.^17^ All binary mixtures were built starting from a perfect crystal of the larger molecule (with size increasing as *sn*-POP < *sn*-POSt < *sn*-StOSt). In all cases, an 800-molecule crystal consisting of a single TAG type was built by stacking 10, 2, and 10 unit cells in the *a*, *b*, and *c* directions, respectively. A specified number of molecules was then selected and replaced at random using a custom Python script (see Supporting Information S.1). The number of molecules replaced was chosen to give crystals consisting of 10–90% of the replacing TAG in the binary TAG crystal in steps of 10%. Every binary crystal made from the same two TAGs, and consisting of the same ratio of TAGs, was built individually, i.e., no two binary TAG crystals were identical.

### Creating Voids

Using the perfect binary TAG crystals, “crack” voids were generated to more closely represent reality and facilitate the melting onset. The description of these types of voids is given in our previous work.^18^ In all cases, the ratio of TAGs was kept to within ±1.5% (absolute) of the starting ratio in the perfect crystal, e.g., if the perfect crystal consisted of 10% *sn*-POSt and 90% *sn*-StOSt, then all the crystals with a crack void had a ratio of *sn*-POSt:*sn*-StOSt of 8.5–11.5%:91.5–88.5%. Void generation was carried out in an automated fashion using a custom Python script (see Supporting Information S.1). Given that each starting perfect binary TAG crystal was different and the void generation was also randomized, no two void systems were identical.

### Melting Point Determination

The determination of the melting point of any binary TAG system was determined using a combination of methodologies described in previous work.^18,22^ For any given simulation, the near-neighbor occupancy (NNO) was determined for the whole system over the full trajectory.^22^ The NNO cutoff distance between the middle oleic chain bead (bead 5 of the oleic chain) was set to 1.2 nm for all cases. The NNO was then summed for the whole system at each time point, reducing the dimensionality of the data from 3 to 2 and plotting the values versus temperature (assuming a perfectly linear increase in temperature along the whole trajectory, thus allowing for an easy time-to-temperature conversion). The melting onset temperature for the system was then determined by iterating along the NNO sum vs temperature plot and determining the point at which the number of NNO data points being lower than their respective predicted interval (PI) was more than 95% of the NNO data points remaining.^18^ For any given TAG 1:TAG 2 ratio and void size combination, the melting point was determined by a simple arithmetic mean of the melting point onset temperature over 50 simulations (see Supporting Information S.2 for the Python script used for melting point determination).

## Results and Discussion

A previous study to determine the melting point of crystals made up of a single type of TAG had shown that the direct heating method was a suitable procedure when using the COGITO FF.^18^ Having defects, which in this case are introduced as “cracks” within the system, be it a TAG system or otherwise, has been shown to be very important to avoid superheating of the system and thus avoid overestimation of the melting point. The presence of voids lowers the estimated melting point due to a lowering of the melting nucleation energy until a plateau is reached with increasing void size.^18,23−29^ While the proportion of the size of the defect to the crystal size is orders of magnitude larger than that observed in real crystals, this methodology has been shown to be both simple and effective. A more in-depth discussion on this can be found in our previous paper and elsewhere.^18,25^ Plotting the average melting onset temperature versus the void size in the crystal shows a clear decrease in the average melting point with increasing void size until a plateau is reached, followed by a sharp decrease in the melting point at larger void sizes, indicating the mechanical collapse of the system, with the melting point before the drop taken to be the melting point of the system.^18,25^ This methodology was tested on five different TAGs, including the TAGs being used in this study, with the simulated melting points being in very good agreement with the empirical melting points.^18^

In this study, a similar methodology was employed to try and determine whether the known nonlinear behavior of binary TAG systems can be reproduced. By creating systems with a mixture of two TAGs, creating a crack void while retaining the ratio of TAGs as close as possible to that in the perfect crystal and heating this system, melting could be observed. In this case, there were two main differences from Cordina et al.,^18^ namely, the reduced number of simulations used per TAG 1:TAG 2 ratio plus void size system (“TAG ratio/void size combination”) and a modification to the melting onset temperature determination for each individual system.

The number of simulations for each TAG ratio/void size combination was reduced from 150 to 50 due to the computational expense involved. Binary combinations of all three TAGs used in this study, ranging from 10 to 90% of a TAG in a system in steps of 10%, with void sizes ranging from 16 to 80 molecules in steps of 8 molecules, result in 12,150 simulations when carrying out 50 simulations for each TAG ratio/void size combination. On inspection of the changing bootstrap error and average melting point with a number of simulation plots, these were determined to be sufficiently converged after 50 runs (see Supporting Information S.3–S.5). In addition, whereas in our previous study, the melting point onset for each simulation was determined from a plot of potential energy versus temperature,^18^ in this study, the melting point onset was determined using the system’s total NNO instead. We also investigated using the change in volume to determine the melting point onset; however, this metric proved unreliable (see Supporting Information S.6).

The NNO is a quick method to determine whether the same molecules are within a cutoff distance of a given reference molecule in subsequent frames in an MD trajectory. If the same molecules are indeed found to be within this cutoff distance in subsequent frames, this indicates that the reference molecule is in a crystalline state, given the relatively short cutoff distance and the unchanging surrounding molecules, as shown previously.^22^ The NNO is calculated based on the central oleic bead, whose distance from the same beads on surrounding molecules should only change if the ordered crystalline structure is lost, except for the expected minor fluctuations. In this case, the more robust nature of NNO was judged to be a better metric than the potential energy metric for binary systems.

**Figure 2:**
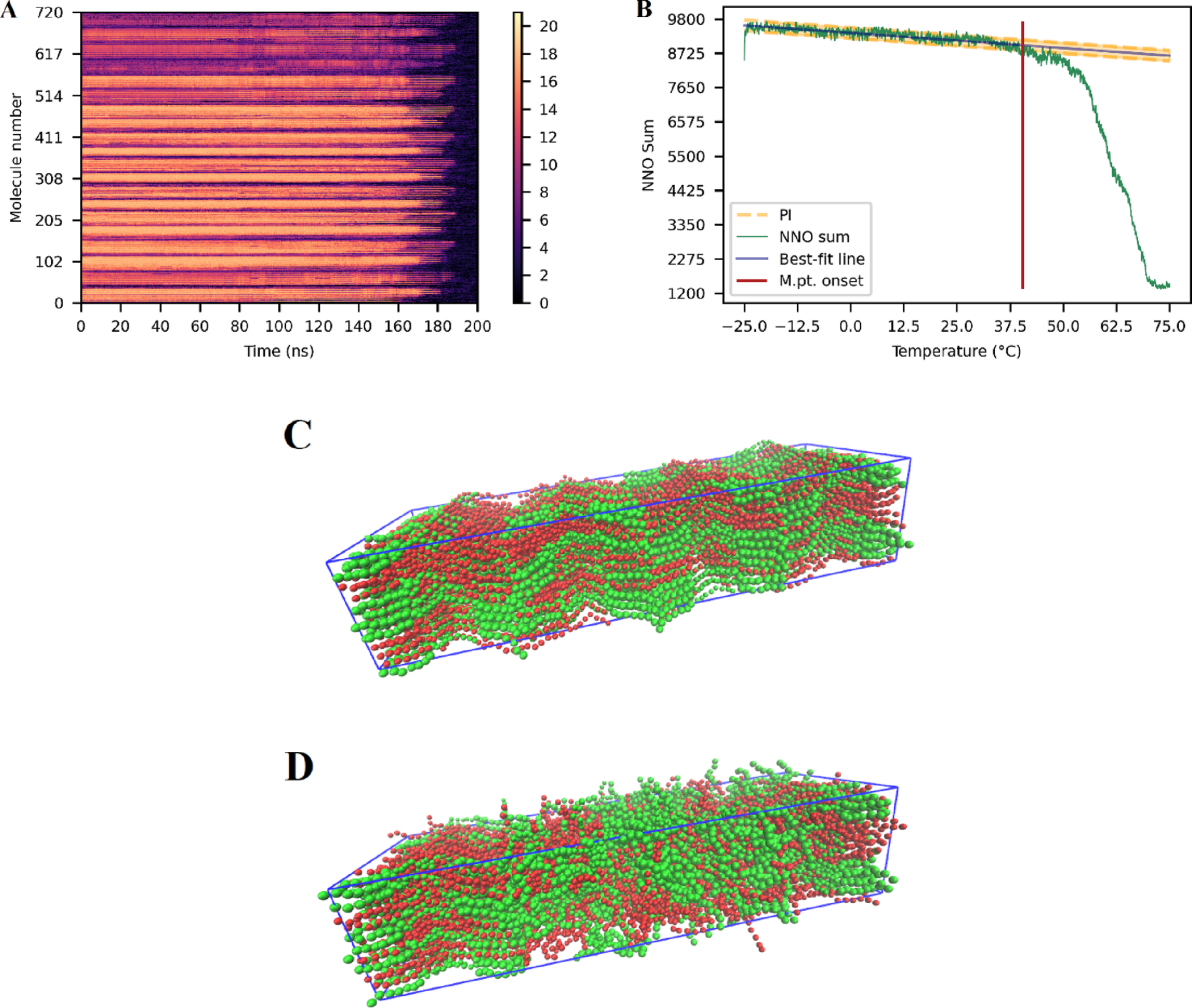
(A) Near-neighbor occupancy plot. The sidebar shows the number of molecules around a given reference molecule. (B) Plot of sum of NNO vs temperature (°C). (C) Minimized (fully crystalline) system (red beads = *sn*-POSt molecules; green beads = *sn*-StOSt molecules). (D) System after 180 ns (most of the system is melted, while parts are still crystalline). All plots are for 50% *sn*-POP/50% *sn*-StOSt with an 80-molecule crack void (run 10).

While the NNO plot (e.g., Figure 2A) gives an easily interpretable visual of the crystalline state or, otherwise, of any given molecule within a system, identifying the exact point at which melting starts requires a rigorous methodology. For this, the methodology using potential energy^18^ was adapted by summing up the NNOs of all the molecules in the system for each frame, plotting these values versus temperature, and then determining the melting point using the lower predicted interval (PI).

As can be seen in Figure 2, the system is very stable for over half of the simulation, as shown by minimal changes in the NNO plot (Figure 2A), and is also reflected in the stable value for the total NNO (Figure 2B). However, toward the end of the simulation, the system starts to change, observed by a marked decrease in the system’s total NNO, which was determined to be the point of melting onset. In the case given in Figure 2, the NNO plot shows that by the end of the simulation, most of the crystal is melted out (darker region of the right-hand side of Figure 2A and a big drop in the total NNO in Figure 2B). This was confirmed by visual inspection of the system at the start (Figure 2C) and after 180 ns (Figure 2D) of the simulation. This gradual melting out of a system was observed previously^18^ and was attributed to the much faster heating rate of MD simulations when compared to an empirical measurement.

**Figure 3:**
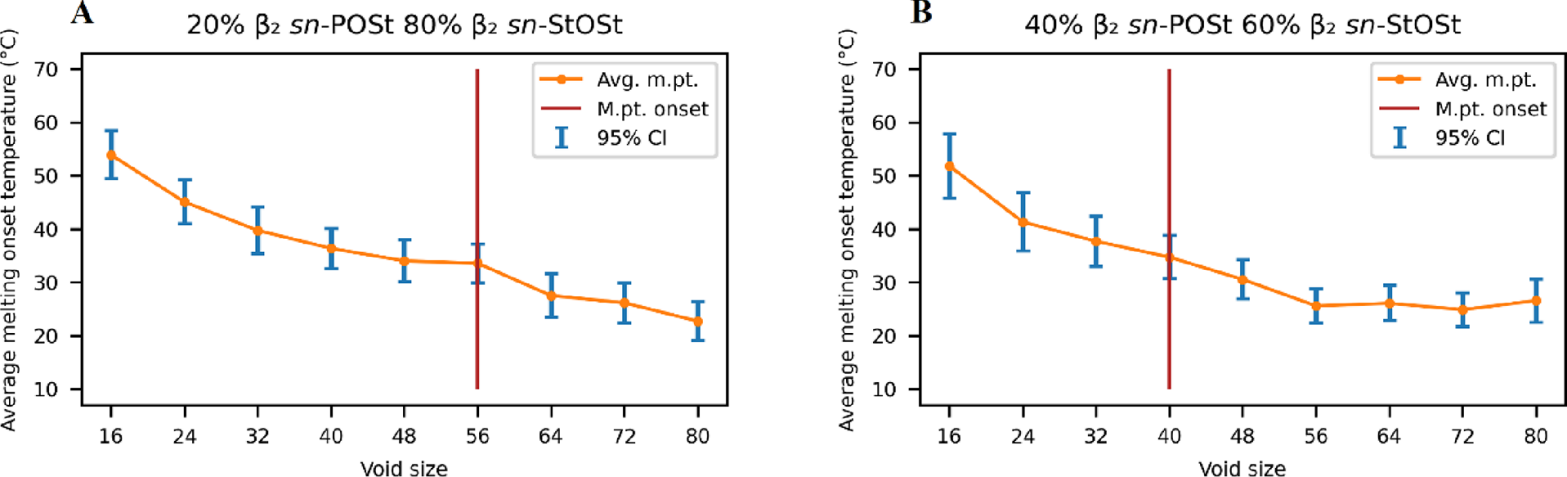
Plots of average melting onset temperature vs void size for (A) 20/80 *sn*-POSt/*sn*-StOSt and (B) 40/60 *sn*-POSt/*sn*-StOSt. Orange dots/line represents the average melting onset temperature. Blue error bars are the 95% confidence interval. The red line indicates the void size/melting point onset for the system.

When plotting the average melting point onset versus the void size for each binary TAG system, a curve can be observed (see Supporting Information S.3–S.5 for all plots); however, the plateau and subsequent drop were not always as clear as for single TAG crystals.^18^ Taking the 20/80 and 40/60 *sn*-POSt/*sn*-StOSt systems as examples (Figure 3), in the former case, the smooth decrease in the average melting onset temperature with increasing void size is very clear, with a plateau reached at a void size of 56 molecules, followed by a clear drop in the melting point (Figure 3A). In the second case, the plateau (or the melting point onset for that binary TAG system) was determined to be the point at which the difference between the melting points at subsequent void sizes was larger than the previous one. In this case, in the steps from a 16- to 40-molecule void, the difference in melting temperature decreases or remains the same, while on going from a 40- to 48-molecule void, the difference in melting temperature increases, giving a discontinuous curve (Figure 3B). This principle was used in all various TAG ratio/void size combinations.

Of all three binary TAG combinations, the average melting onset temperature versus void size curves of the *sn*-POSt/*sn*-StOSt (see Supporting Information S.3) were the most difficult to interpret. Taking the 30/70 *sn*-POSt/*sn*-StOSt system as an example, the correct void size is not immediately obvious, as there is a marked decrease in melting temperature on going from a 24- to 32-molecule void, which would indicate that the melting point at a 24-molecule void is the melting point for this system. However, when looking at the plot as a whole and taking the other *sn*-POSt/*sn*-StOSt systems into consideration, the determined melting temperature at a 32-molecule void size seems to not follow the trend. Going from a 40- to 64-molecule void, the curve is very smooth, while when looking at the very smooth 20/80 *sn*-POSt/*sn*-StOSt system, the plateau is clearly reached at a 56-molecule void, i.e., at a much larger void size than 24 molecules. Taking this into consideration, the melting point for the 30/70 *sn*-POSt/*sn*-StOSt system was thus determined to be at a 64-molecule void. A similar approach was taken for the 80/20 *sn*-POSt/*sn*-StOSt system. Given this, the determined melting points for each TAG ratio/void size combination are listed in Table 1.

**Table 1:** Simulated Melting Point (°C) for All TAG Ratios for All Binary Systems^a^

TAG ratio	*sn*-POP/*sn*-POSt	*sn*-POP/*sn*-StOSt	*sn*-POSt/*sn*-StOSt
0/100	34.4	38.9	38.9
10/90	28.5	43.5	30.0
20/80	26.4	33.8	33.6
30/70	20.5	30.4	30.4
40/60	22.4	28.9	34.7
50/50	18.8	28.8	33.6
60/40	21.1	23.6	31.4
70/30	23.2	19.5	24.9
80/20	24.4	15.6	20.4
90/10	28.3	16.3	26.9
100/0	33.5	33.5	34.4

a Simulated temperatures for 0/100 and 100/0 ratio systems, i.e., for the pure TAGs, obtained from Cordina et al.^18^

**Figure 4:**
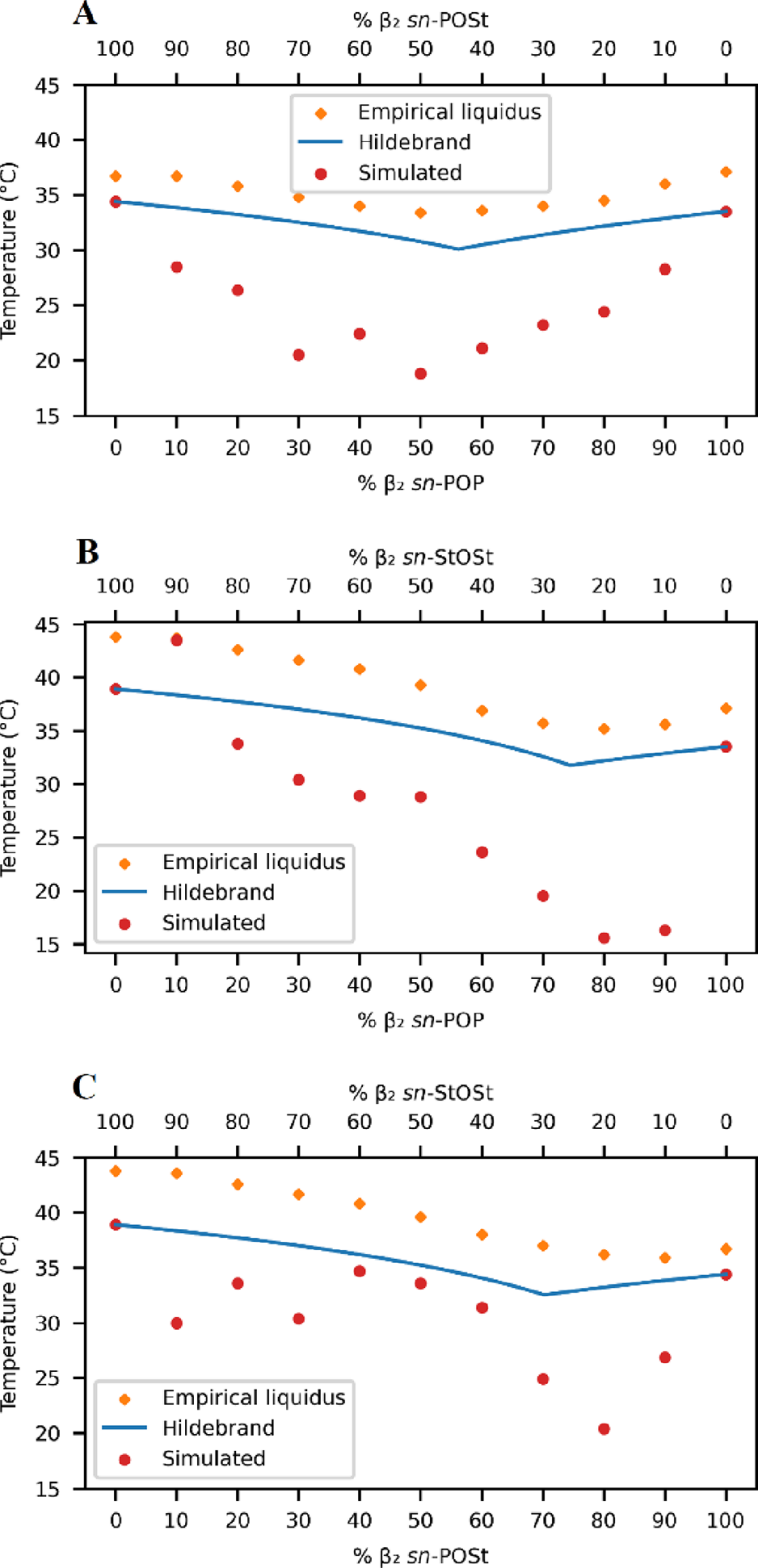
Phase diagrams of binary TAG systems. (A) β_2_*sn*-POP/ β_2_*sn*-POSt; (B) β_2_*sn*-POP/ β_2_*sn*-StOSt; (C) β_2_*sn*-POSt/ β_2_*sn*-StOSt. Orange diamonds indicate empirical liquidus temperatures.^10^ The solid blue line indicates predicted liquidus.^16^ Red dots are the simulated melting temperature.

Plotting these values, and comparing them to empirical measurements^10^ and predicted values from the Hildebrand model,^10,16^ shows good trend reproduction (Figure 4). The empirical measurements were approximated from Smith et al.,^10^ and they show the temperature at 5% solid. The predicted liquidus line from the Hildebrand model was calculated using the simulated melting points of the pure TAGs^18^ and enthalpies of fusion obtained from the literature.^30,31^

All three phase diagrams clearly show the nonlinear mixing behavior of the binary TAG systems, whether from the empirical measurements, the predicted phase diagram, or the simulated temperatures from this study. It is, however, observed that the simulated melting points of the binary systems are generally lower than the empirical or predicted values (Figure 4). However, the general trend is reproduced well. Taking the *sn*-POP/*sn*-POSt binary system as an example, a minimum at around 50/50 ratio can be clearly observed (Figure 4A), confirming that the eutectic behavior of this binary system is observed during the simulations.

**Figure 5:**
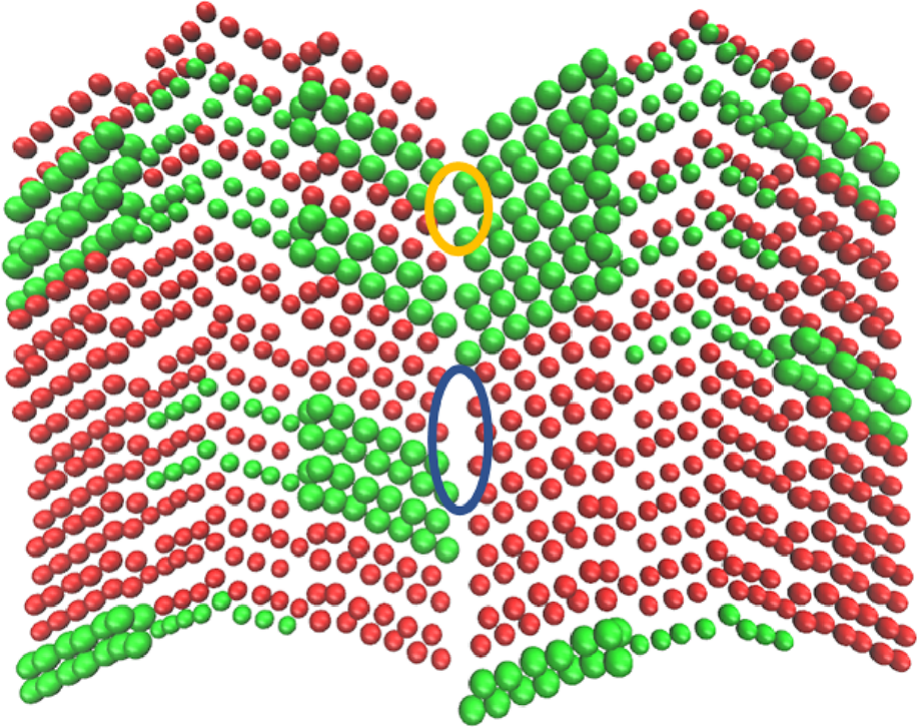
Cross section of a 50% *sn-*POP (red beads)/50% *sn*-StOSt (green beads) crystal. The yellow circle shows the area with the ends of the stearic chains in close proximity. The blue circle shows the area with mini-voids created by the presence of *sn*-POP in a crystal with *sn*-StOSt.

A possible reason for the lower-than-expected melting points could be extra mini-voids. These are created when, for example, *sn*-StOSt, a longer chain TAG, is in a crystal with *sn*-POP, a shorter chain TAG, with the palmitic fatty acid chain represented by one less bead than the stearic chain in the COGITO FF. This is shown in Figure 5, where one can clearly see more space between the ends of the fatty acid chains where *sn*-POP is present. Given the importance of voids to lower the nucleation melting energy barrier during MD simulations,^18,23−25,27,28^ having an artificially created void (the crack void), along with the mini-voids created due to the differing chain lengths, leads to an amplified lowering of the energy barrier, which results in a lower-than-expected melting point.

## Conclusions

In this study, we have shown the suitability of the COGITO FF to simulate the nonlinear behavior of binary TAG systems. While this study was carried out on known binary systems, being able to reproduce the nonlinear trends of such systems increases the confidence in the prediction of the behavior of unknown binary TAG systems.

The results in this study show that the predicted melting points of the binary systems are lower than expected, possibly as a result of the increased number of mini-voids created by the different TAG chain lengths. Given that the COGITO FF was not specifically parametrized to be able to reproduce melting points of mixed TAG crystals, the ability to correctly identify the binary composition at which the eutectic point is observed is an indication of the broad applicability of this FF. However, this methodology provides a powerful and simple way to determine the nonlinear/eutectic behavior of any binary crystalline TAG system, especially for those where the thermodynamic properties (melting temperature and enthalpy of fusion) are not known and, hence, where mathematical predictive models cannot be used. This study thus provides a methodology to determine binary TAG behavior without any empirical measurements.

## Supporting Information Available

S.1: Python script to generate perfect binary TAG crystals with random distribution of the TAGs, followed by the creation of crack voids; S.2: Python script to determine the melting point of a single system and any given TAG ratio–void size combination; S.3: results for different void sizes for different *sn*-POSt:*sn*-StOSt ratios; S.4: results for different void sizes for different *sn*-POP:*sn*-POSt ratios; S.5: results for different void sizes for different *sn*-POP:*sn*-StOSt ratios; S.6: plots of average melting point onset temperature vs void size (determined using volume change) for different *sn*-POP:*sn*-POSt ratios; S.7: plots of average melting point onset temperature vs void size (determined using volume change) for different *sn*-POSt:*sn*-StOSt ratios; S.8: plots of average melting point onset temperature vs void size (determined using volume change) for different *sn*-POP:*sn*-StOSt ratios (PDF)

## Notes

All bootstrapping error plots, force field files, starting configurations, and topologies used in this study can be found at https://doi.org/10.15129/a3e63cff-c28f-441e-a670-f84b995fcb8e.

## References

[ref1] Stingelin N. On the Phase Behaviour of Organic Semiconductors. Polym. Int. 2012, 61, 866–873. 10.1002/pi.4214.

[ref2] Manara D.; Jackson H. F.; Perinetti-Casoni C.; Boboridis K.; Welland M. J.; Luzzi L.; Ossi P. M.; Lee W. E. The ZrC–C Eutectic Structure and Melting Behaviour: A High-Temperature Radiance Spectroscopy Study. J. Eur. Ceram. Soc. 2013, 33, 1349–1361. 10.1016/j.jeurceramsoc.2012.12.008.

[ref3] Frongia F.; Pilloni M.; Scano A.; Ardu A.; Cannas C.; Musinu A.; Borzone G.; Delsante S.; Novakovic R.; Ennas G. Synthesis and Melting Behaviour of Bi, Sn and Sn–Bi Nanostructured Alloy. J. Alloys Compd. 2015, 623, 7–14. 10.1016/j.jallcom.2014.08.122.

[ref4] Stolarska O.; Rodríguez H.; Smiglak M. Eutectic Mixtures of Pyrrolidinium-Based Ionic Liquids. Fluid Phase Equilib. 2016, 408, 1–9. 10.1016/j.fluid.2015.08.007.

[ref5] Teles Dos Santos M.; Gerbaud V.; Roux Le G A C Solid Fat Content of Vegetable Oils and Simulation of Interesterification Reaction: Predictions from Thermodynamic Approach. J. Food Eng. 2013, 126, 198 10.1016/j.jfoodeng.2013.11.012.

[ref6] Farmani J. Modeling of Solid Fat Content of Chemically Interesterified Fully Hydrogenated Soybean Oil and Canola Oil Blends as a Function of Temperature and Saturated Fatty Acids. J. Food Meas. Charact. 2015, 9, 281–289. 10.1007/s11694-015-9233-8.

[ref7] Augusto P. E. D.; Soares B. M. C.; Chiu M. C.; Gonçalves L. A. G. Modelling the Effect of Temperature on the Lipid Solid Fat Content (SFC). Food Res. Int. 2012, 45, 132–135. 10.1016/j.foodres.2011.10.026.

[ref8] Rousset P.; Rappaz M.; Minner E. Polymorphism and Solidification Kinetics of the Binary System POS-SOS. J. Am. Oil Chem. Soc. 1998, 75, 851 10.1007/s11746-998-0237-y.

[ref9] Sato K. Crystallization Behaviour of Fats and Lipids — a Review. Chem. Eng. Sci. 2001, 56, 2255–2265. 10.1016/S0009-2509(00)00458-9.

[ref10] Smith K. W.; Bhaggan K.; Talbot G. Phase Behavior of Symmetrical Monounsaturated Triacylglycerols. Eur. J. Lipid Sci. Technol. 2013, 115, 838–846. 10.1002/ejlt.201300035.

[ref11] Zhang L.; Ueno S.; Sato K. Binary Phase Behavior of Saturated-Unsaturated Mixed-Acid Triacylglycerols—A Review. J. Oleo Sci. 2018, 67, 679–687. 10.5650/jos.ess17263. 29760333

[ref12] Macridachis-González J.; Bayés-García L.; Calvet A. T. An Insight into the Solid-State Miscibility of Triacylglycerol Crystals. Molecules 2020, 25, 4562 10.3390/molecules25194562. 33036267 PMC7583920

[ref13] Pizzirusso A.; Peyronel F.; Co E. D.; Marangoni A. G.; Milano G. Molecular Insights into the Eutectic Tripalmitin/Tristearin Binary System. J. Am. Chem. Soc. 2018, 140, 12405–12414. 10.1021/jacs.8b04729. 30178998

[ref14] Pizzirusso A.; Brasiello A.; De Nicola A.; Marangoni A. G.; Milano G. Coarse-Grained Modelling of Triglyceride Crystallisation: A Molecular Insight into Tripalmitin Tristearin Binary Mixtures by Molecular Dynamics Simulations. J. Phys. D: Appl. Phys. 2015, 48, 494004 10.1088/0022-3727/48/49/494004.

[ref15] Marangoni A. G.; Wesdorp L. H. Liquid–Multiple Solid Phase Equilibria In Fats: Theory And Experiments. In Structure And Properties Of Fat Crystal Networks;CRC Press, 2013; Pp 260–437.

[ref16] Hildebrand J. H.; Solubility X. I. I. Regular Solutions. J. Am. Chem. Soc. 1929, 51, 66–80. 10.1021/ja01376a009.

[ref17] Cordina R. J.; Smith B.; Tuttle T. COGITO: A Coarse-Grained Force Field for the Simulation of Macroscopic Properties of Triacylglycerides. J. Chem. Theory Comput. 2023, 19, 1333–1341. 10.1021/acs.jctc.2c0097. 36728833 PMC9979597

[ref18] Cordina R. J.; Smith B.; Tuttle T. Triacylglyceride Melting Point Determination using Coarse-Grained Molecular Dynamics. J. Comput. Chem. 2023, 44, 1795 10.1002/jcc.27128. 37163230

[ref19] Sasaki M.; Ueno S.; Sato K. 6 - Polymorphism And Mixing Phase Behavior Of Major Triacylglycerols Of Cocoa Butter. In Cocoa Butter And Related Compounds; Elsevier Inc., 2012; pp 151–172.

[ref20] Van Der Spoel D.; Lindahl E.; Hess B.; Groenhof G.; Mark A. E.; Berendsen H. J. C. GROMACS: Fast, Flexible, and Free. J. Comput. Chem. 2005, 26, 1701–1718. 10.1002/jcc.20291. 16211538

[ref21] Peschar R.; Schenk H.; van Mechelen J. B. Structures of Mono-unsaturated Triacylglycerols. II. the β2 Polymorph. Acta Crystallogr., Sect. B: Struct. Sci., Cryst. Eng. Mater. 2006, 62, 1131–1138. 10.1107/S0108768106037074. 17108668

[ref22] Cordina R. J.; Smith B.; Tuttle T. Rapid Automated Quantification Of Triacylglyceride Crystallinity In Molecular Dynamics Simulations. J. Chem. Inf. Model. 2022, 62, 5601–5606. 10.1021/acs.jcim.2c00972. 36332114 PMC9709910

[ref23] Agrawal P. M.; Rice B. M.; Thompson D. L. Molecular Dynamics Study Of The Effects Of Voids And Pressure In Defect-Nucleated Melting Simulations. J. Chem. Phys. 2003, 118, 9680–9688. 10.1063/1.1570815.

[ref24] Agrawal P. M.; Rice B. M.; Thompson D. L. Molecular Dynamics Study Of The Melting Of Nitromethane. J. Chem. Phys. 2003, 119, 9617–9627. 10.1063/1.1612915.

[ref25] Eike D. M.; Brennecke J. F.; Maginn E. J. Toward A Robust And General Molecular Simulation Method For Computing Solid-Liquid Coexistence. J. Chem. Phys. 2005, 122, 14115 10.1063/1.1823371. 15638650

[ref26] Zhang S. L.; Zhang X. Y.; Qi L.; Wang L. M.; Zhang S. H.; Zhu Y.; Liu R. P. The Study of Melting Stage of Bulk Silicon using Molecular Dynamics Simulation. Physica. B 2011, 406, 2637–2641. 10.1016/j.physb.2011.04.005.

[ref27] Gavezzotti A. A Molecular Dynamics View Of Some Kinetic And Structural Aspects Of Melting In The Acetic Acid Crystal. J. Mol. Struct. 1999, 485–486, 485 10.1016/S0022-2860(99)00056-3.

[ref28] Orekhov N. D.; Stegailov V. V. Molecular Dynamics Simulation Of Graphite Melting. High Temp. 2014, 52, 198–204. 10.1134/S0018151X14020187.

[ref29] Zou Y.; Xiang S.; Dai C. Investigation on the Efficiency and Accuracy of Methods for Calculating Melting Temperature by Molecular Dynamics Simulation. Comput. Mater. Sci. 2020, 171, 109156 10.1016/j.commatsci.2019.109156.

[ref30] Sato K.; Arishima T.; Wang Z. H.; Ojima K.; Sagi N.; Mori H. Polymorphism Of POP And SOS. I. Occurrence And Polymorphic Transformation. J. Am. Oil Chem. Soc. 1989, 66, 664–674. 10.1007/BF02669949.

[ref31] Arishima T.; Sagi N.; Mori H.; Sato K. Polymorphism Of POS. I. Occurrence And Polymorphic Transformation. J. Am. Oil Chem. Soc. 1991, 68, 710–715. 10.1007/BF02662157.

