# Supplementary material for: Predicting Lipid Eutectics Using Coarse-Grained Molecular Dynamics
Source: J Phys Chem B. 2023 Nov 17;127(47):10236–42. doi: 10.1021/acs.jpcb.3c06297 (PMC10694813; doi:10.1021/acs.jpcb.3c06297)

# Supporting Information

# Predicting Lipid Eutectics Using Coarse-Grained Molecular Dynamics

*Robert J. Cordina,*[1,2] *Beccy Smith,*[1] *Tell Tuttle*[2,*]

[1] Cadbury UK Ltd., PO Box 12, Bournville Lane, Birmingham B30 2LU, UK.

[2] Department of Pure and Applied Chemistry, University of Strathclyde, 295 Cathedral Street, Glasgow G1 1XL, UK.

* tell.tuttle@strath.ac.uk +44 141 548 2290

S.1 – Python script to generate perfect binary TAG crystals with random distribution of the TAGs, followed by the creation of crack voids

```
#!/usr/bin/env python3
# -*- coding: utf-8 -*-

import numpy as np
import pandas as pd
import os
import subprocess
import time

starting_tag = 'SOS'        # perfect pure crystal TAG type; SOS or POS
replacing_tag = 'POS'       # TAG to be used to replaced in perfect starting_tag crystal; POS or POP

replace_percent = [10,20,30,40,50,60,70,80,90]      # % of replacing_TAG in starting_TAG crystal to create a perfect mixed crystal
percentage_voids = [1,2,3,4,5,6,7,8,9,10]           # % molecules to be removed from mixed crystal (create void)

replacement_type = 'random'
void_type = 'cracks'


crystal_size = 800          # number of molecules in perfect starting_tag crystal

num_iterations = 150        # number of perfect crystals with replaced TAGs to generate (equivalent to number of runs)

binary_mixture = replacing_tag + '_' + starting_tag  # replacing_tag in starting_tag



# dictionary used to convert numeric to alpha
num_to_word = {
    1: 'one',
    2: 'two',
    3: 'three',
    4: 'four',
    5: 'five',
    6: 'six',
    7: 'seven',
    8: 'eight',
    9: 'nine',
    10:'ten',
    11:'eleven',
    12:'twelve',
    13:'thirteen',
    14:'fourteen',
    15:'fifteen',
    16:'sixteen',
    17:'seventeen',
    18:'eighteen',
    19:'nineteen',
    20:'twenty',
    25:'twentyfive',
    30:'thirty',
    35:'thirtyfive',
    40:'forty',
    45:'fortyfive',
    50:'fifty',
    55:'fiftyfive',
    60:'sixty',
    65:'sixtyfive',
    70:'seventy',
    75:'seventyfive',
    80:'eighty',
    85:'eightyfive',
    90:'ninety',
    95:'ninetyfive',
    100: 'hundred'    }


def molecules_to_be_replaced(percent, crystal_size):
    # determining how many molecules are to be replaced
    replace_mols = int(percent/100 * crystal_size)

    # determining which molecules are to be replaced, chosen randomly
    mols_to_replace = np.array([])

    while len(mols_to_replace) < replace_mols:
        random_num = np.random.randint(low=1, high=crystal_size+1)          # '+ 1' is required as np.random.randint works on [low,high) basis
        if random_num not in mols_to_replace:
            mols_to_replace = np.append(mols_to_replace, random_num)

    # splitting the molecule numbers into evens and odds, as these have mirror configurations in space in the crystal, and hence require a
different replacing TAG configuration
    odd_mols = np.array([])
    even_mols = np.array([])

    for mols in mols_to_replace:
        if mols%2 == 0:
            even_mols = np.append(even_mols,mols)
        else:
            odd_mols = np.append(odd_mols,mols)

    return odd_mols, even_mols


def replace_molecules(odd_mols, even_mols):
    '''
    Writing gro file with replaced molecules automatically using GROMACS.
    This is done by writing the positions.dat file with the Centre of Geometry coordinates
    of the molecules to be replaced, and then writing and running a bash script with the
    GROMACS insert-molecules command with the -replace flag.
    '''

    # this file has to be generated manually by the user and placed in the folder
    # e.g. if it's a POP in SOS blend, then folder name is POP_SOS,
    # and if crystal_size is 800, then file name is SOS-800mols.gro
    file = wdir2 + starting_tag + '-' + str(crystal_size) + 'mols.gro'
```

```
    # get all bead data from gro file and place into a DataFrame
    df = data_extraction(file)

    # determining the Centre of Geometry of the molecules to be replaced in the crystal
    odd_mols_coords = n.zeros(shape=(len(odd_mols),3))
    even_mols_coords = np.zeros(shape=(len(even_mols),3))

    for num in range(len(odd_mols)):
        odd_mols_coords[num][0] = (df.loc[df.res_num == odd_mols[num], 'x']).mean()
        odd_mols_coords[num][1] = (df.loc[df.res_num == odd_mols[num], 'y']).mean()
        odd_mols_coords[num][2] = (df.loc[df.res_num == odd_mols[num], 'z']).mean()

    for num in range(len(even_mols)):
        even_mols_coords[num][0] = (df.loc[df.res_num == even_mols[num], 'x']).mean()
        even_mols_coords[num][1] = (df.loc[df.res_num == even_mols[num], 'y']).mean()
        even_mols_coords[num][2] = (df.loc[df.res_num == even_mols[num], 'z']).mean()

    # set (and make if not present) sub-directory 2 with percentage of replacing_tag
    folder = num_to_word[percent] + '_per_' + replacing_tag + '/'

    if os.path.isdir(wdir2 + folder) != True:
        os.makedirs(wdir2 + folder)

    # writing the positions.dat file, used by GROMACS in the gmx insert-molecules command to direct
    # exactly where the replacing molecules are to be placed, based on the CoG determination in the step above
    with open(wdir2 + folder + 'positions.dat', 'w') as f:
        for num in range(len(odd_mols)):
            for i in range(3):
                coord = str(odd_mols_coords[num,i])
                f.write(coord + '\t')
            f.write('\n')

    # writing a short bash script with the GROMACS command to replace the randomly chosen molecules, and executing it
    with open(wdir + 'replacement_script.sh' ,'w') as f:
        f.write('#!/bin/bash' + '\n')
        f.write('source /usr/local/gromacs/bin/GMXRC' + '\n')
        f.write('cd ' + wdir2 + folder + '\n')
        f.write('gmx insert-molecules -f ' + file + ' -o box2.gro -ci ../../../../../' + replacing_tag + '-mol1.gro -rot none -ip -nmol ' +
str(len(odd_mols)) + ' -replace')

    process = subprocess.run(wdir + 'answers.sh', shell=True, check=True, timeout=240)

    # process is repeated for evenly-numbered molecules, due to different configuration in space of odd and evenly numbered molecules
    with open(wdir2 + folder + 'positions.dat', 'w') as f:
        for num in range(len(even_mols)):
            for i in range(3):
                coord = str(even_mols_coords[num,i])
                f.write(coord + '\t')
            f.write('\n')

    with open(wdir + 'replacement_script.sh' ,'w') as f:
        f.write('#!/bin/bash' + '\n')
        f.write('source /usr/local/gromacs/bin/GMXRC' + '\n')
        f.write('cd ' + wdir2 + folder + '\n')
        f.write('gmx insert-molecules -f box2.gro -o box3.gro -ci ../../../../../' + replacing_tag + '-mol2.gro -rot none -ip -nmol ' +
str(len(even_mols)) + ' -replace')

    process = subprocess.run(wdir + 'answers.sh', shell=True, check=True, timeout=240)


def write_gro_file(output_file, df, xx, yy, zz, xy, xz, yx, yz, zx, zy, system_name):
    with open(output_file, "w") as outfile:

        # write binary mixture name and percentage replacement
        # outfile.write(str(num_to_word[percent] + ' percent ' + replacing_tag + ' in ' + starting_tag))
        outfile.write(str(system_name))
        outfile.write(str('\n'))

        # write total number of beads
        outfile.write(str('   ') + str(int(len(df))))
        outfile.write(str('\n'))

        # get data to be written
        textdata = df.iloc[:,:3].to_numpy()
        numdata = df.iloc[:,3:].to_numpy()

        for j in range(len(df)):

            # bead index number; from 1 to total number of beads; resetting to 1 after 99999
            if (j+1 <= 99999):
                number = j+1
            elif (j+1 > 99999):
                number = j-99999

            # determine spacing before residue number
            if int(textdata[j,0]) < 10:
                space0 = '    '
            elif 10 <= int(textdata[j,0]) <= 99:
                space0 = '   '
            elif 100 <= int(textdata[j,0]) <= 999:
                space0 = '  '
            elif 1000 <= int(textdata[j,0]) <= 9999:
                space0 = ' '

            # determine spacing before consecutive bead number
            if number < 10:
                space1 = '    '
            elif 10 <= number <= 99:
                space1 = '   '
            elif 100 <= number <= 999:
                space1 = '  '
            elif 1000 <= number <= 9999:
                space1 = ' '
            elif 10000 <= number <= 99999:
                space1 = ''

            # determine spacing before coordinate values (one less space if a '-' sign is present)
            if numdata[j,0] >= 0 and numdata[j,0] < 10:
                space2 = '   '
            else:
                space2 = '  '

            if numdata[j,1] >= 0 and numdata[j,1] < 10:
```

```
                space3 = '   '
            else:
                space3 = '  '

            if numdata[j,2] >= 0 and numdata[j,2] < 10:
                space4 = '   '
            else:
                space4 = '  '


            # writing each atom/bead and position
            outfile.write(  space0  + str(textdata[j,0]) +                          # residue number
                                      str(textdata[j,1]) +                          # TAG type
                            str('   ') + str(textdata[j,2]) +                       # bead tyoe
                            str(space1)  + str(int(number)) +                       # index number
                            str(space2)  + str("{:.3f}".format(numdata[j,0])) +     # x-coordinate
                            str(space3)  + str("{:.3f}".format(numdata[j,1])) +     # y-coordinate
                            str(space4)  + str("{:.3f}".format(numdata[j,2]))   )   # z-coordinate
            outfile.write(str('\n'))

        # writing box dimensions and vector angles
        outfile.write(str('    ') +
                      str("{:.5f}".format(xx)) +
                      str('   ') +
                      str("{:.5f}".format(yy)) +
                      str('    ') +
                      str("{:.5f}".format(zz)) +
                      str('    ') +
                      str("{:.5f}".format(xy)) +
                      str('    ') +
                      str("{:.5f}".format(xz)) +
                      str('    ') +
                      str("{:.5f}".format(yx)) +
                      str('    ') +
                      str("{:.5f}".format(yz)) +
                      str('    ') +
                      str("{:.5f}".format(zx)) +
                      str('    ') +
                      str("{:.5f}".format(zy)) )


def data_extraction(file):
    # count number of lines in mixed crystal gro file
    with open(file, 'r') as f:
        number_of_lines = 0
        for line in f:
            number_of_lines += 1

        # create empty DataFrame to store information of substituted box
        colNames = ['res_num', 'TAG', 'bead_name', 'x', 'y', 'z']

        df = pd.DataFrame(index=range(number_of_lines-3),columns=range(len(colNames)))
        df.columns = colNames

        for data_capt in range(6):

            if data_capt == 0:
                temp_data = np.ndarray(shape=(number_of_lines-3,1),dtype=int)
            elif data_capt == 1 or data_capt == 2:
                temp_data = np.ndarray(shape=(number_of_lines-3,1)).astype(str)
            else:
                temp_data = np.ndarray(shape=(number_of_lines-3,1),dtype=float)

            # extract residue number, TAG type, bead name and coordinate data of all beads and place in DataFrame
            f.seek(0)
            count = 0
            for line in f:
                count += 1
                if 2 < count < number_of_lines:
                    if data_capt == 0:
                        temp_data[count-3,0] = line[0:5]
                    elif data_capt == 1:
                        temp_data[count-3,0] = str(line[5:8])
                    elif data_capt == 2:
                        temp_data[count-3,0] = str(line[11:15])
                    if data_capt == 3:
                        temp_data[count-3,0] = line[21:28]
                    if data_capt == 4:
                        temp_data[count-3,0] = line[28:36]
                    if data_capt == 5:
                        temp_data[count-3,0] = line[36:44]

            df.iloc[:,data_capt] = temp_data

    df.reset_index(drop=True, inplace=True)

    return df


def box_geometry(starting_tag, crystal_size):

    if starting_tag == 'POS':
        if crystal_size == 800:
            x = 5.4243
            y = 25.3058
            z = 8.1206
        if crystal_size == 8000:
            x = 10.84860
            y = 63.26450
            z = 16.23580

        alpha = 90
        beta = 88.51
        gamma = 90

    elif starting_tag == 'SSS':
        x = 12.0053
        y = 20.7608
        z = 5.4450
        alpha = 73.75
        beta = 100.260002
        gamma = 117.690002
```

```
    elif starting_tag == 'SOS':
        if crystal_size == 800:
            x = 5.4401001
            y = 26.0589996
            z = 8.2208557
        elif crystal_size == 100:
            x = 2.72005
            y = 13.02950
            z = 4.10945
        alpha = 90
        beta = 88.750259
        gamma = 90

    elif starting_tag == 'POP':
        x = 5.4471001
        y = 24.3244003
        z = 8.2195633
        alpha = 90
        beta = 88.780045
        gamma = 90

    elif starting_tag == 'COC':
        x = 5.4471001
        y = 16.3165207
        z = 8.2195633
        alpha = 90
        beta = 88.780045
        gamma = 90

    # box vector calculations based on box lengths and angles

    v  = ((1 - np.cos(np.radians(alpha))**2 - np.cos(np.radians(beta))**2 - np.cos(np.radians(gamma))**2 + 2 * np.cos(np.radians(alpha)) *
np.cos(np.radians(beta)) * np.cos(np.radians(gamma))) ** 0.5) * x * y * z

    xx = x
    yy = y * np.sin(np.radians(gamma))
    zz = v / (x * y * np.sin(np.radians(gamma)))
    xy = 0
    xz = 0
    yx = y * np.cos(np.radians(gamma))
    yz = 0
    zx = z * np.cos(np.radians(beta))
    zy = (z/np.sin(np.radians(gamma))) * (np.cos(np.radians(alpha)) - np.cos(np.radians(beta)) * np.cos(np.radians(gamma)))

    return xx, yy, zz, xy, xz, yx, yz, zx, zy


def residue_renumbering_and_sorting(file):

    # get all bead data
    # returns DataFrame containing residue number, TAG type, bead name, xyz coordinates
    df = data_extraction(file)

    # section only entered if the residue numbers are not from 1 to crystal_size
    if df.iloc[0,0] != 1 and df.iloc[-1,0] != crystal_size:

        # extract residue numbers list from DataFrame
        resnr = df.iloc[:,0].to_numpy()
        # extract TAG type of all beads from DataFrame
        TAG_list = (df.iloc[:,1].to_numpy()).astype(str)

        rescount = 1
        beadcount = 0

        # go through each bead to determine whether the residue number and the TAG type are sequential
        while rescount < crystal_size:
            while beadcount < len(resnr):

                if TAG_list[beadcount] == 'POP':
                    beadnum = 20
                elif TAG_list[beadcount] == 'POS':
                    beadnum = 21
                elif TAG_list[beadcount] == 'SOS':
                    beadnum = 22

                # if the residue number is not correct, then this is changed to the correct one, to end up with a list from 1 to crystal_size
                if resnr[beadcount] != rescount:
                    resnr[beadcount:beadcount+beadnum] = rescount
                    rescount += 1
                    beadcount += beadnum

        # DataFrame is updated with new residue numbers
        df.iloc[:,0] = resnr


    # change residue numbers to reflect original numbers
    # e.g. if starting_tag residue 10 had been chosen at random at the start of the script, and this had been
    # replaced with a different TAG, which in turn was automatically given a new residue number by GROMACS, then the
    # residue number of the replacing_tag is changed back to the original starting_tag residue number
    # so that the order of the molecules in the crystal is preserved
    all_subsituted_mols = np.append(odd_mols,even_mols)

    replacing_tag_res_numbers = np.array([])

    for asm in range(len(all_subsituted_mols)):

        if replacing_tag == 'POP':
            replacing_tag_res_numbers = np.append(replacing_tag_res_numbers,[all_subsituted_mols[asm]]*20)
        elif replacing_tag == 'POS':
            replacing_tag_res_numbers = np.append(replacing_tag_res_numbers,[all_subsituted_mols[asm]]*21)
        elif replacing_tag == 'SOS':
            replacing_tag_res_numbers = np.append(replacing_tag_res_numbers,[all_subsituted_mols[asm]]*22)

    df.iloc[(len(df)-len(replacing_tag_res_numbers)):,0] = replacing_tag_res_numbers

    # change renumbered starting_tag residue numbers (automatically renumbered by GROMACS during the molecule replacement command)
    # to their original residue numbers to preserve the same order of residue numbers in the original crystal
    sorted_all_subsituted_mols = np.sort(all_subsituted_mols)
    starting_tag_res_numbers = np.array([])

    counter = 1
    while counter <= crystal_size:
        if starting_tag == 'POP':
```

```
            number_of_beads = 20
        elif starting_tag == 'POS':
            number_of_beads = 21
        elif starting_tag == 'SOS':
            number_of_beads = 22

        if counter not in sorted_all_subsituted_mols:
            starting_tag_res_numbers = np.append(starting_tag_res_numbers,[counter]*number_of_beads)
        counter += 1

    df.iloc[:len(starting_tag_res_numbers),0] = starting_tag_res_numbers

    # sort DataFrame in order of the residue number
    df = df.sort_values(by='res_num', ascending=True, ignore_index=True, kind='stable')
    df['res_num'] = df['res_num'].astype('int')     # used to make sure that numbers are integers and not floats, e.g., 1 and not 1.0
    df['res_num'] = df['res_num'].astype('str')     # convert to string to facilitate extraction along with TAG type to numpy array

    return df


def create_voids():

    # extract all bead data from mixed, no void, crystal
    file = wdir2 + folder + num_to_word[percent] + '_per_' + replacing_tag + '_' + starting_tag + '_run' + str(iteration+1) + '.gro'
    df = data_extraction(file)

    # drop all duplicate residue number + TAG name combos
    df_no_duplicates = df.drop_duplicates(subset=['res_num', 'TAG'], keep='last')

    # array to store whether TAG type is replacing_tag or starting_tag
    mol_type_list = np.zeros(shape=(crystal_size))

    # fill mol_type_list
    for i in range(crystal_size):
        if df_no_duplicates.iloc[i,1] == starting_tag:
            mol_type_list[i] = 1

    # number of molecules in x, y and directions in a perfect crystal (no voids)
    x_axis = 8
    y_axis = 10
    z_axis = 10

    # array to store molecule nubers as per crystal structure
    res_ids = np.zeros(shape=(x_axis,y_axis,z_axis))

    # array to store molecule type in mixed crystal; 1 ==  starting_tag
    tag_type_array = np.zeros(shape=(x_axis,y_axis,z_axis)).astype(int)

    # numbers of 1st line of molecules (x-dimension)
    a,b,c,d,e,f,g,h = 1,4,3,2,41,44,43,42

    for i in range(y_axis):
        for j in range(z_axis):
                res_ids[0,i,j] = a + i*4 + j*80
                res_ids[1,i,j] = b + i*4 + j*80
                res_ids[2,i,j] = c + i*4 + j*80
                res_ids[3,i,j] = d + i*4 + j*80
                res_ids[4,i,j] = e + i*4 + j*80
                res_ids[5,i,j] = f + i*4 + j*80
                res_ids[6,i,j] = g + i*4 + j*80
                res_ids[7,i,j] = h + i*4 + j*80

                tag_type_array[0,i,j] = mol_type_list[a + i*4 + j*80 -1]
                tag_type_array[1,i,j] = mol_type_list[b + i*4 + j*80 -1]
                tag_type_array[2,i,j] = mol_type_list[c + i*4 + j*80 -1]
                tag_type_array[3,i,j] = mol_type_list[d + i*4 + j*80 -1]
                tag_type_array[4,i,j] = mol_type_list[e + i*4 + j*80 -1]
                tag_type_array[5,i,j] = mol_type_list[f + i*4 + j*80 -1]
                tag_type_array[6,i,j] = mol_type_list[g + i*4 + j*80 -1]
                tag_type_array[7,i,j] = mol_type_list[h + i*4 + j*80 -1]


    # percent_count used to determine whether it's the first percentage in the list or not,
    # and hence whether script should start from a random molecule or not - this allows for
    # the voids to "build on each other"
    percent_count = 0

    # iterate over void sizes for each mixed crystal
    for percentage_void in range(len(percentage_voids)):

        # calculate total number of molecules in perfect crystal
        num_of_mols = x_axis * y_axis * z_axis
        # percentage of moleules to remove from crystal
        percent_to_remove = percentage_voids[percentage_void]
        # calculate number of molecules to remove from crystal
        mols_to_remove = int(percent_to_remove / 100 * num_of_mols)

        if void_type == 'cracks':

            if percent_count == 0:
                # choose initial seed molecule index
                seed_x = np.random.randint(0,x_axis)
                seed_y = np.random.randint(0,y_axis)
                seed_z = np.random.randint(0,z_axis)

                # array with indices of molecules to remove; all initially set to -1
                indices_to_remove = np.zeros(shape=(mols_to_remove,3))
                indices_to_remove[:,:] = -1

                # start populating array with seed molecule index
                indices_to_remove[0,0] = seed_x
                indices_to_remove[0,1] = seed_y
                indices_to_remove[0,2] = seed_z

                # inititate counter of molecules removed so far
                count = 0

            if percent_count > 0:   # this is the percent from the percentage_voids list
                # set counter of molecules removed so far
                count = len(indices_to_remove)-1

                # create array to store new molecules to remove
                additional_indices = np.zeros(shape=((mols_to_remove-len(indices_to_remove),3)))
```

```
                additional_indices[:,:] = -1
                # add the additional "spaces" to indices_to_remove
                indices_to_remove = np.vstack((indices_to_remove,additional_indices))


            while count < mols_to_remove-1:

                # array with indices of all molecules to be removed so far
                mols = indices_to_remove[indices_to_remove[:,0] != -1, :]

                # empty array to store index of new molecule to be removed
                new_coords = np.ndarray(shape=3)

                # select -1, 0 or 1 at random to determine direction of next molecule to be removed
                x = np.random.randint(-1,2)     # np.random.randint chooses a number from [x,y), hence 2 is never chosen
                y = np.random.randint(-1,2)
                z = np.random.randint(-1,2)

                # choose one molecule at random from list of molecules to be removed so far
                if len(mols) == 1:
                    anchor_mol = 0
                else:
                    anchor_mol = np.random.randint(0,len(mols))

                # calculate index of new molecule to be removed based on random anchor molecule and random direction
                new_coords[0] = indices_to_remove[anchor_mol,0] + x
                new_coords[1] = indices_to_remove[anchor_mol,1] + y
                new_coords[2] = indices_to_remove[anchor_mol,2] + z

                # check if next identified molecule index is already in the list of molecules to remove - avoids duplicates
                if (new_coords==mols[:,None]).all(-1).any():
                    count += 0
                elif x_axis-1 >= new_coords[0] >= 0 and y_axis-1 >= new_coords[1] >= 0 and z_axis-1 >= new_coords[2] >= 0:
                    count += 1
                    indices_to_remove[count,:] = new_coords
                else:
                    count += 0


        # covert all molecules indices to integers
        indices_to_remove = indices_to_remove.astype(int)

        # empty array to store mapped indices to molecule numbers
        mol_nums = np.ndarray(shape=mols_to_remove).astype(int)

        # find and store mapped molecule numbers from indices
        for i in range(len(mol_nums)):
            mol_nums[i] = res_ids[indices_to_remove[i,0],indices_to_remove[i,1],indices_to_remove[i,2]]

        percent_count += 1


        # extract all bead data from mixed crystal
        file = wdir2 + folder + num_to_word[percent] + '_per_' + replacing_tag + '_' + starting_tag + '_run' + str(iteration+1) + '.gro'
        df = data_extraction(file)


        # remove all molecules which have been chosen at random to create voids
        df = df[~df.res_num.isin(mol_nums)]
        df.reset_index(drop=True, inplace=True)

        # extract TAG names sequence from DataFrame
        TAG_sequence = df.drop_duplicates(subset=['res_num', 'TAG'], keep='last')

        # find out how many TAGs are of which type
        # group by TAG type
        number_of_TAGs = TAG_sequence.groupby(['TAG'])['TAG'].count()

        TAG_counts[replacement_counter,iteration,percentage_void] = number_of_TAGs[replacing_tag] / (number_of_TAGs[replacing_tag] +
number_of_TAGs[starting_tag]) * 100


        # convert TAG_sequence DataFrame to numpy array
        TAG_sequence = TAG_sequence.to_numpy().astype(str)

        # renumber all residues
        tag_res_numbers = np.array([])

        tag_res_num_counter = 0
        for Ts in range(len(TAG_sequence)):

            tag_res_num_counter += 1

            if TAG_sequence[Ts,1] == 'POP':
                tag_res_numbers = np.append(tag_res_numbers,[tag_res_num_counter]*20).astype(int)
            elif TAG_sequence[Ts,1] == 'POS':
                tag_res_numbers = np.append(tag_res_numbers,[tag_res_num_counter]*21).astype(int)
            elif TAG_sequence[Ts,1] == 'SOS':
                tag_res_numbers = np.append(tag_res_numbers,[tag_res_num_counter]*22).astype(int)

        df['res_num'] = tag_res_numbers

        # get box size and angles
        xx, yy, zz, xy, xz, yx, yz, zx, zy = box_geometry(starting_tag, crystal_size)

        # write gro file
        if os.path.isdir(wdir2 + folder + '/' + num_to_word[percentage_voids[percentage_void]] + '_percent_void') != True:
            os.makedirs(wdir2 + folder + '/' + num_to_word[percentage_voids[percentage_void]] + '_percent_void')

        if type(percent_to_remove) == int:
            gro_file = (wdir2 + folder + '/' + num_to_word[percentage_voids[percentage_void]] + '_percent_void' + '/'
                        + replacing_tag + '_' + str(percent) + '_' + starting_tag + '_' + str(int(percent_to_remove)) + 'per_run' +
str(int(iteration + 1)) + '.gro' )
            top_file = (wdir2 + folder + '/' + num_to_word[percentage_voids[percentage_void]] + '_percent_void' + '/'
                        + replacing_tag + '_' + str(percent) + '_' + starting_tag + '_' + str(int(percent_to_remove)) + 'per_run' +
str(int(iteration + 1)) + '.top' )
        else:
            gro_file = (wdir2 + folder + '/' + num_to_word[percentage_voids[percentage_void]] + '_percent_void' + '/'
                        + replacing_tag + '_' + str(percent) + '_' + starting_tag + '_' + str(percent_to_remove) + 'per_run' +
str(int(iteration + 1)) + '.gro' )
            top_file = (wdir2 + folder + '/' + num_to_word[percentage_voids[percentage_void]] + '_percent_void' + '/'
                        + replacing_tag + '_' + str(percent) + '_' + starting_tag + '_' + str(percent_to_remove) + 'per_run' +
str(int(iteration + 1)) + '.top' )
```

```
        system_name = str(num_to_word[percent] + ' percent ' + replacing_tag + ' in ' + starting_tag + ' '
                          + str(percentage_voids[percentage_void]) + '% percent void'
                          + ' run ' + str(iteration+1))
        write_gro_file(gro_file, df, xx, yy, zz, xy, xz, yx, yz, zx, zy, system_name)

        # create topology files
        topology_generator(gro_file, top_file, percentage_voids[percentage_void])

        gro_activate(gro_file)


        ## use only for checking void size and position

        if iteration <= 10:

            # extract all bead data from mixed, no void, crystal
            file = wdir2 + folder + num_to_word[percent] + '_per_' + replacing_tag + '_' + starting_tag + '_run' + str(iteration+1) + '.gro'
            full_coords = data_extraction(file)


            gro_file = (wdir2 + folder + '/' + num_to_word[percentage_voids[percentage_void]] + '_percent_void' + '/'
                              + replacing_tag + '_' + str(percent) + '_' + starting_tag + '_' + str(int(percent_to_remove)) + 'per_run' +
str(int(iteration + 1)) + '.gro' )
            with_voids_coords = data_extraction(gro_file)


            voids_file = (wdir2 + folder + '/' + num_to_word[percentage_voids[percentage_void]] + '_percent_void' + '/'
                              + replacing_tag + '_' + str(percent) + '_' + starting_tag + '_' + str(int(percent_to_remove)) + 'per_run' +
str(int(iteration + 1)) + '_voids.gro' )


            voids_coords = pd.merge(full_coords, with_voids_coords, how='outer',indicator=True, on =['x', 'y', 'z'])
            voids_coords = voids_coords[(voids_coords._merge != 'both')]
            voids_coords = voids_coords.drop(['_merge'], axis=1)

            write_gro_file(voids_file, voids_coords, xx, yy, zz, xy, xz, yx, yz, zx, zy, system_name)
            gro_activate(voids_file)


def topology_generator(gro_file, top_file, percentage_void):

    df = data_extraction(gro_file)

    TAG_sequence = df.drop_duplicates(subset=['res_num', 'TAG'], keep='last')

    TAG_sequence.insert(2, "sum", 0)

    counter = 1
    for i in range(len(TAG_sequence)):

        if i == len(TAG_sequence)-1:
            TAG_sequence.iloc[i,2] = counter

        elif TAG_sequence.iloc[i,1] == TAG_sequence.iloc[i+1,1]:
            counter += 1

        else:
            TAG_sequence.iloc[i,2] = counter
            counter = 1

    TAG_sequence = TAG_sequence[TAG_sequence["sum"] != 0]
    TAG_sequence = TAG_sequence.iloc[:,1:3]

    with open(top_file, 'w') as f:

        f.write('#include "COGITO.ff/forcefield.itp"')
        f.write('\n'*2)

        f.write('#include "../../../' + TAG_sequence.iloc[0,0] + '-singlemol.top"')
        f.write('\n')
        if TAG_sequence.iloc[0,1] > 1:
            f.write('[ molecules ]')
            f.write('\n')
            f.write(TAG_sequence.iloc[0,0]+ '\t' + str(int(TAG_sequence.iloc[0,1]-1)))
            f.write('\n')
            f.write('#include "../../../' + TAG_sequence.iloc[1,0] + '-singlemol.top"')
            f.write('\n')

        else:
            f.write('#include "../../../' + TAG_sequence.iloc[1,0] + '-singlemol.top"')
            f.write('\n')

        if TAG_sequence.iloc[1,1] > 1:
            f.write('[ molecules ]')
            f.write('\n')
            f.write(TAG_sequence.iloc[1,0]+ '\t' + str(int(TAG_sequence.iloc[1,1]-1)))
            f.write('\n')

        for Tn in range(len(TAG_sequence)-2):
            f.write(TAG_sequence.iloc[Tn+2,0] + '\t' + str(TAG_sequence.iloc[Tn+2,1]))
            f.write('\n')


def fix_coordinates(file):

    with open(wdir + 'fix.sh', 'w') as f:
        f.write('#!/bin/bash' + '\n')
        f.write('sed -i "s/-0.000/0.000/" ' + file + '\n')

    process = subprocess.run(wdir + 'fix.sh', shell=True, check=True, timeout=240)


def gro_activate(file):

    with open(wdir + 'fix.sh', 'w') as f:
        f.write('#!/bin/bash' + '\n')
        f.write('file="' + file + '"' + '\n')
        f.write('echo "   " >> $file')

    process = subprocess.run(wdir + 'fix.sh', shell=True, check=True, timeout=240)
```

```
# parent directory
wdir = '/path/to/dir/'

# sub-directory 1 specifying which binary mixture; format is replaing_tag in starting_tag, e.g., POP_SOS means POP is replaced SOS crystal
wdir2 = wdir + binary_mixture + '/' + replacement_type + '_replacement/' + void_type + '/'

if os.path.isdir(wdir2) != True:
    os.makedirs(wdir2)

# create empty array to store how much of each TAG are in the final boxes after void creation
TAG_counts = np.ndarray(shape=(len(replace_percent),num_iterations,len(percentage_voids)))

replacement_counter = -1
# iterating though the percentage of molecules to be replaced in the perfect pure TAG crystal
for percent in replace_percent:

    replacement_counter += 1

    # sub-directory 2 specifying the percentage of replacing_tag in the perfect pure starting_tag crystal
    folder = num_to_word[percent] + '_per_' + replacing_tag + '/'

    # iterating through the specified number of iterations/different systems to be generated (not void sizes)
    for iteration in range(num_iterations):

        # determine how many and which molecules are to be replaced
        odd_mols, even_mols = molecules_to_be_replaced(percent, crystal_size)


        # replace the randomly chosen molecules in the perfect pure starting_tag crystal using GROMACS and write the resulting gro files
        # ends up with a file called box3.gro
        replace_molecules(odd_mols, even_mols)

        # residue renumbering and sorting of box3.gro
        file = wdir2 + folder + 'box3.gro'
        df = residue_renumbering_and_sorting(file)

        # get box size and angles
        xx, yy, zz, xy, xz, yx, yz, zx, zy = box_geometry(starting_tag, crystal_size)

        # specify file name for perfect mixed TAG crystal
        output_file = wdir2 + folder + num_to_word[percent] + '_per_' + replacing_tag + '_' + starting_tag + '_run' + str(iteration+1) + '.gro'
        top_file = wdir2 + folder + num_to_word[percent] + '_per_' + replacing_tag + '_' + starting_tag + '_run' + str(iteration+1) + '.top'

        # write gro file for perfect mixed TAG crystal, using the renumbered and sorted box3.gro data
        system_name = str(num_to_word[percent] + ' percent ' + replacing_tag + ' in ' + starting_tag + ' run ' + str(iteration+1))
        write_gro_file(output_file, df, xx, yy, zz, xy, xz, yx, yz, zx, zy, system_name)

        # fix any -0.000 errors
        fix_coordinates(output_file)

        # create topology files
        topology_generator(output_file, top_file, percent)

        # activate perfect crystal gro files
        gro_activate(output_file)

        # remove GROMACS-generated files
        os.remove(wdir2 + folder + 'box2.gro')
        os.remove(wdir2 + folder + 'box3.gro')


        # choose which molecules to remove to create void and generate gro files
        # iterating over as many void sizes as specified in percentage_voids
        # create_voids()

    # write percentage of replacing_tag in the system after void creation
    np.savetxt(wdir2 + folder + 'overall_TAG_nums.csv', TAG_counts[replacement_counter,:,:], fmt='%.2f')
```

S.2 – Python script to determine the melting point of a single system, and any given TAG ratio-void size combination

```
#!/usr/bin/env python3
# -*- coding: utf-8 -*-

import time
import re
import numpy as np

import matplotlib.pyplot as plt
from mpl_toolkits.axes_grid1 import make_axes_locatable

from numba import jit as njit
from numba import prange

from mdtraj import load_xtc as xtc

from scipy.stats import linregress, bootstrap

import warnings
warnings.filterwarnings('ignore', category=UserWarning)

from numba.core.errors import NumbaPerformanceWarning
warnings.simplefilter('ignore', category=NumbaPerformanceWarning)


@njit(parallel=True, nopython=True)
def cutoff_indices_func(num_frames,
                        mol_count,
                        cutoff_indices,
                        coordinates):
    """
    Function determining which molecules (molecule indices) are within 1.2 nm away from the reference molecule
    for each molecule in each frame.

    The if statement in the inner for loop avoids the calculation of the distance between the same molecule
    by using the ref != num conditional.

    """

    for frame in prange(num_frames):
        for ref in prange(mol_count):
            for num in prange(mol_count):

                if (ref != num and
                      ( ( (coordinates[frame,ref,0] - coordinates[frame,num,0])**2 +
                          (coordinates[frame,ref,1] - coordinates[frame,num,1])**2 +
                          (coordinates[frame,ref,2] - coordinates[frame,num,2])**2 ) ** 0.5 <= 1.2) ):
                    cutoff_indices[frame, ref, num] = 1


@njit(parallel=True, nopython=True)
def same_indices(num_frames,
                 mol_count,
                 same_indices_count,
                 cutoff_indices):
    """
    Function determining whether a molecule found to be within the cut-off distance of the reference molecule
    is also found within the cut-off distance in the subseqent frame.

    """

    for frame in prange(num_frames):
        for ref in prange(mol_count):
            for num in prange(mol_count):
                # determine which molecules are common between frames for each molecules
                if frame != num_frames-1:
                    if cutoff_indices[frame,ref,num] == 1 and cutoff_indices[frame,ref,num] == cutoff_indices[frame+1,ref,num]:
                        same_indices_count[frame,ref,num] = 1


@njit(parallel=True, nopython=True)
def count_same_indices(num_frames, mol_count, same_indices_copy, same_indices_count):
    for frame in prange(num_frames):
        for ref in prange(mol_count):
            same_indices_count[frame,ref] = np.sum(same_indices_copy[frame,ref,:])


@njit(parallel=True, nopython=True)
def nno_count(num_frames, total_nno, same_indices_count_transposed):
    for frame in prange(num_frames):
        total_nno[frame] = same_indices_count_transposed[:,frame].sum()


def nno_plot(same_indices_count_transposed):
    """
    heatmap showing how many molecules within the cut-off distance for one molecule are the same in
    subsequent frames for each molecule over time

    """

    fig = plt.figure(figsize=(3.33,2.25), dpi=450)
    ax = fig.add_subplot(111)

    im = ax.imshow(same_indices_count_transposed, aspect='auto', cmap=plt.cm.magma, extent=[0,total_traj_time,mol_count,0])
    ax.invert_yaxis()

    ax.set_xlabel('Time (ns)', fontsize=6)
    ax.set_ylabel('Molecule number', fontsize=6)
    # ax.set_title('Near neighbour occupancy', fontsize=10)
    plt.xticks(np.linspace(0, total_traj_time, int((total_traj_time/20)+1)), fontsize=6)

    plt.yticks(np.linspace(0, mol_count, 8).astype(int), fontsize=6)

    divider = make_axes_locatable(ax)
    cax = divider.append_axes("right", size="2%", pad=0.2)
    cbar = plt.colorbar(im, cax=cax, ticks=np.linspace(0,20,11))
    cbar.ax.tick_params(labelsize=6)
```

```
    plt.show()

    fig.savefig(wdir + 'nno.jpg', format='jpg')


def nno_sum_plot(total_nno, temperatures, nno_lower_PI, nno_upper_PI, melting_onset):

    fig = plt.figure(figsize=(3.33,2.25), dpi=450)
    ax = fig.add_subplot(111)

    ax.plot(temperatures,nno_lower_PI, color='orange', linestyle='dashed', alpha=0.5, zorder=1)
    ax.plot(temperatures,nno_upper_PI, color='orange', linestyle='dashed', alpha=0.5, zorder=1, label='PI')
    ax.fill_between(temperatures[start:],nno_lower_PI[start:],nno_upper_PI[start:], color ='orange',  alpha=0.3, zorder=1)
    ax.plot(temperatures, total_nno, label='NNO sum', zorder=2, color='seagreen')
    ax.plot(temperatures,nno_predict,color='navy', alpha=0.5, label='Best-fit line', zorder=3, linewidth=1)
    ax.vlines(melting_onset, min(total_nno), max(total_nno), color='firebrick', label='M.pt. onset', zorder=4)

    ax.set_xlabel('Temperature (°C)', fontsize=6)
    ax.set_ylabel('NNO Sum', fontsize=6)

    plt.xticks(np.linspace(-25,75,9), fontsize=6)
    plt.yticks(fontsize=6)

    ax.legend(fontsize=6)

    plt.show()

    fig.savefig(wdir + 'nno_sum.jpg', format='jpg')



start_time = time.time()


starting_tag = 'SOS'        # SOS or POS
replacing_tag = 'POS'       # POS or POP


wdir = '/path/to/dir/binary_mixtures/' + replacing_tag + '_' + starting_tag + '/random_replacement/cracks/'


replacements_alpha = ['ten', 'twenty', 'thirty', 'forty', 'fifty', 'sixty', 'seventy', 'eighty', 'ninety']
replacements_num = [10,20,30,40,50,60,70,80,90]
voids_alpha = ['two', 'three', 'four', 'five', 'six', 'seven', 'eight', 'nine', 'ten']
voids_num = [2,3,4,5,6,7,8,9,10]

start_iteration = 1
end_iteration = 50



np.set_printoptions(linewidth=np.inf)

for rep in range(len(replacements_alpha)):

    for void in range(len(voids_alpha)):

        melting_onset = np.zeros(shape=(int(end_iteration-start_iteration+1)))

        for run in np.linspace(start_iteration,
                               end_iteration,
                               int(end_iteration-start_iteration+1)
                               ).astype(int):

            file_text = (wdir +
                         replacements_alpha[rep] + '_per_' + replacing_tag + '/' +
                         voids_alpha[void] + '_percent_void/' +
                         replacing_tag + '_' + str(replacements_num[rep]) + '_' + starting_tag + '_' + str(voids_num[void]) + 'per_run' +
str(run) )

            min_file = file_text + '_min.gro'
            traj_file = file_text + '_eq.xtc'
            log_file = file_text + '_eq.log'


            # opening the log file, searching for the timestep, number of steps and frame capture interval lines,
            # extracting the values, converting them to a float and thus calculating the total trajectory time and
            # number of frames in the trajectory
            with open(log_file, 'r') as f:
                for line in f:
                    if 'dt' in line and '=' in line:                        # finding the 'dt' line
                        dt = float(re.findall("\d+\.\d+", line)[0])         # getting the timestep in ps
                    if 'nsteps' in line and '=' in line:                    # finding the 'nsteps' line
                        nsteps = float(re.findall("\d+\\d+", line)[0])      # getting the number of steps
                        total_traj_time = nsteps * dt / 1000                # calculating the total trajectory time in ns
                        break                                               # breaks the loop to avoid reading the whole file

            # determining the number of molecules in the simulation box
            mol_count = len(np.unique(np.genfromtxt(min_file, usecols=0, dtype=str, skip_header=2, skip_footer=1)))

            # finding which beads are the middle oleic bead in each molecule; indices will be 1 less due to 0-indexing
            O5_list = []

            with open(min_file, 'r') as f:
                lines = f.readlines()
                lines = lines[2:-1]

            for line in range(len(lines)):
                if 'O2B5' in lines[line]:
                    O5_list.append(line)

            # extracting coordinates for all beads for all the trajectory using MDTraj
            traj = xtc(traj_file,
                       top = min_file)
            # placing all the coordinates in a 3D numpy array with the shape=(num_frames,mol_count*bead_count,(x,y,z))
            coordinates = traj.xyz
            coordinates = coordinates[:2001,O5_list,:]
            coordinates = np.ascontiguousarray(coordinates)

            num_frames = len(coordinates)

            if total_traj_time > 200:
```

```
            total_traj_time = 200

        # determine which molecules are within the cut-off distance of each molecule
        cutoff_indices = np.ndarray(shape=(num_frames,mol_count,mol_count), dtype=np.int32)
        # Set all values of array to 0
        cutoff_indices[:,:,:] = 0

        cutoff_indices_func(num_frames,
                            mol_count,
                            cutoff_indices,
                            coordinates)


        # determine near neighbour occupancy in subsequent frames
        same_indices_count = np.ndarray(shape=(num_frames,mol_count,mol_count), dtype=np.int32)
        # Set all values of array to 0
        same_indices_count[:,:,:] = 0

        same_indices(num_frames,
                     mol_count,
                     same_indices_count,
                     cutoff_indices)

        # setting the values of the last frame equal to that those of the penultimate frame
        same_indices_count[-1,:,:] = same_indices_count[-2,:,:]


        same_indices_copy = same_indices_count
        same_indices_count = np.zeros(shape=(num_frames,mol_count))
        count_same_indices(num_frames, mol_count, same_indices_copy, same_indices_count)

        same_indices_count_transposed = np.transpose(same_indices_count)

        nno_plot(same_indices_count_transposed)



        total_nno = np.zeros(shape=num_frames)

        nno_count(num_frames, total_nno, same_indices_count_transposed)


        # determine melting point onset
        start = int(len(total_nno)*0.05)
        end = int(len(total_nno)*0.3)
        PI = 1.96   # 1.96=95%, 2.57=99%

        temperatures = np.linspace(-25,75,len(total_nno))

        nno_gradient = linregress(temperatures[start:end],total_nno[start:end])[0]
        nno_intercept = linregress(temperatures[start:end],total_nno[start:end])[1]
        nno_predict = nno_gradient * temperatures + nno_intercept
        nno_sum_errs = np.sum((nno_predict[start:end]-total_nno[start:end])**2)
        nno_stdev = (1/(len(total_nno[start:end])-2) * nno_sum_errs)**0.5
        nno_interval = PI * nno_stdev
        nno_lower_PI = nno_predict - nno_interval
        nno_upper_PI = nno_predict + nno_interval


        for i in range(len(total_nno)):
            if ( (total_nno[i:] < nno_lower_PI[i:]).sum() > 0.95 * len(nno_lower_PI[i:]) and
                i > start):
                melting_onset[run-1] = temperatures[i]
                break
            else:
                melting_onset[run-1] = max(temperatures)


        nno_sum_plot(total_nno, temperatures, nno_lower_PI, nno_upper_PI, melting_onset[run-1])


        del(cutoff_indices)
        del(same_indices_count)
        del(same_indices_copy)
        del(same_indices_count_transposed)
        del(coordinates)
        del(traj)
        del(total_nno)
        del(lines)



    num_iter = end_iteration-start_iteration+1
    bootstrap_steps = num_iter-10+1
    err_array = np.ndarray(shape=(bootstrap_steps))
    means = np.ndarray(shape=(bootstrap_steps))
    st_dev = np.ndarray(shape=(bootstrap_steps))

    try:
        count = 0
        for j in np.linspace(10,num_iter,bootstrap_steps).astype(int):
            data_onset = (melting_onset[:j],)
            res_onset = bootstrap(data_onset, np.mean, confidence_level=0.95, n_resamples=10000, random_state=np.random.default_rng())

            err_array[count] = res_onset.standard_error
            means[count] = (res_onset.confidence_interval[1] + res_onset.confidence_interval[0])/2
            st_dev[count] = len(data_onset)**0.5 * (res_onset.confidence_interval[1] - res_onset.confidence_interval[0]) / 3.92

            count += 1

    except ValueError:
        print('Bootstrapping error')

    print(replacements_num[rep], '%', replacing_tag, voids_num[void], '% void',
          'Avg melting point =', round(melting_onset.mean(),2),
          '95% CI:', str(round(1.96 * np.std(melting_onset) / (bootstrap_steps**0.5),2)),'\t',
          'Bootstrap melting onset:', round( (res_onset.confidence_interval[0] + res_onset.confidence_interval[1])/2, 2))

print('This script took',
      '{:.2f}'.format((time.time()-start_time)), 'seconds or',
      '{:.2f}'.format((time.time()-start_time)/60), 'minutes or',
      '{:.2f}'.format((time.time()-start_time)/3600), 'hours', '\n')
```

## S.3 – Results for Different Void Sizes for Different *sn*-POSt : *sn*-StOSt ratios

*Table S.3a - Average Melting Onset Temperature*

| | % *sn*-POSt | | | | | | | | |
|---|---|---|---|---|---|---|---|---|---|
| void size (molecules) | 10% | 20% | 30% | 40% | 50% | 60% | 70% | 80% | 90% |
| 16 | 50.17 | 53.94 | 52.41 | 51.86 | 48.14 | 48.83 | 47.32 | 50.94 | 45.36 |
| 24 | 49.32 | 45.09 | 50.00 | 41.35 | 41.63 | 40.77 | 41.98 | 34.67 | 35.52 |
| 32 | 41.18 | 39.76 | 37.12 | 37.72 | 38.17 | 34.27 | 34.74 | 35.96 | 29.00 |
| 40 | 34.80 | 36.38 | 37.72 | 34.74 | 33.60 | 31.43 | 28.77 | 26.68 | 26.89 |
| 48 | 30.03 | 34.04 | 33.61 | 30.55 | 27.21 | 25.47 | 26.59 | 22.91 | 21.43 |
| 56 | 29.99 | 33.58 | 31.40 | 25.59 | 24.73 | 26.88 | 24.38 | 21.19 | 20.82 |
| 64 | 27.50 | 27.53 | 30.41 | 26.09 | 23.25 | 21.18 | 24.88 | 20.41 | 20.90 |
| 72 | 26.20 | 26.16 | 24.19 | 24.86 | 21.15 | 23.34 | 20.65 | 18.36 | 18.29 |
| 80 | 25.75 | 22.73 | 23.88 | 26.59 | 19.19 | 21.84 | 19.22 | 15.88 | 16.93 |

*Table S.3b - 95% Confidence Interval*

| | % *sn*-POSt | | | | | | | | |
|---|---|---|---|---|---|---|---|---|---|
| void size (molecules) | 10% | 20% | 30% | 40% | 50% | 60% | 70% | 80% | 90% |
| 16 | 6.19 | 4.50 | 4.42 | 6.00 | 5.62 | 4.95 | 5.54 | 4.74 | 5.72 |
| 24 | 3.97 | 4.12 | 4.99 | 5.43 | 4.50 | 4.12 | 3.94 | 4.49 | 4.74 |
| 32 | 4.42 | 4.31 | 3.68 | 4.70 | 4.14 | 3.29 | 4.07 | 3.56 | 3.40 |
| 40 | 4.40 | 3.77 | 4.12 | 4.03 | 3.57 | 3.07 | 3.69 | 2.79 | 3.49 |
| 48 | 4.28 | 3.96 | 4.15 | 3.65 | 3.26 | 3.23 | 3.03 | 2.80 | 2.98 |
| 56 | 4.13 | 3.63 | 4.17 | 3.18 | 3.92 | 3.17 | 3.49 | 2.63 | 2.70 |
| 64 | 3.70 | 4.06 | 3.82 | 3.27 | 2.82 | 3.30 | 3.27 | 2.90 | 2.86 |
| 72 | 4.32 | 3.73 | 3.50 | 3.17 | 3.20 | 3.32 | 2.60 | 2.40 | 2.64 |
| 80 | 3.88 | 3.67 | 2.96 | 4.05 | 2.73 | 3.04 | 2.92 | 2.38 | 3.67 |

*Figure S.3a-3i Plots of Average Melting Onset Temperature vs Void Size for mixtures of $\beta_2$ sn-POSt and sn-StOSt*

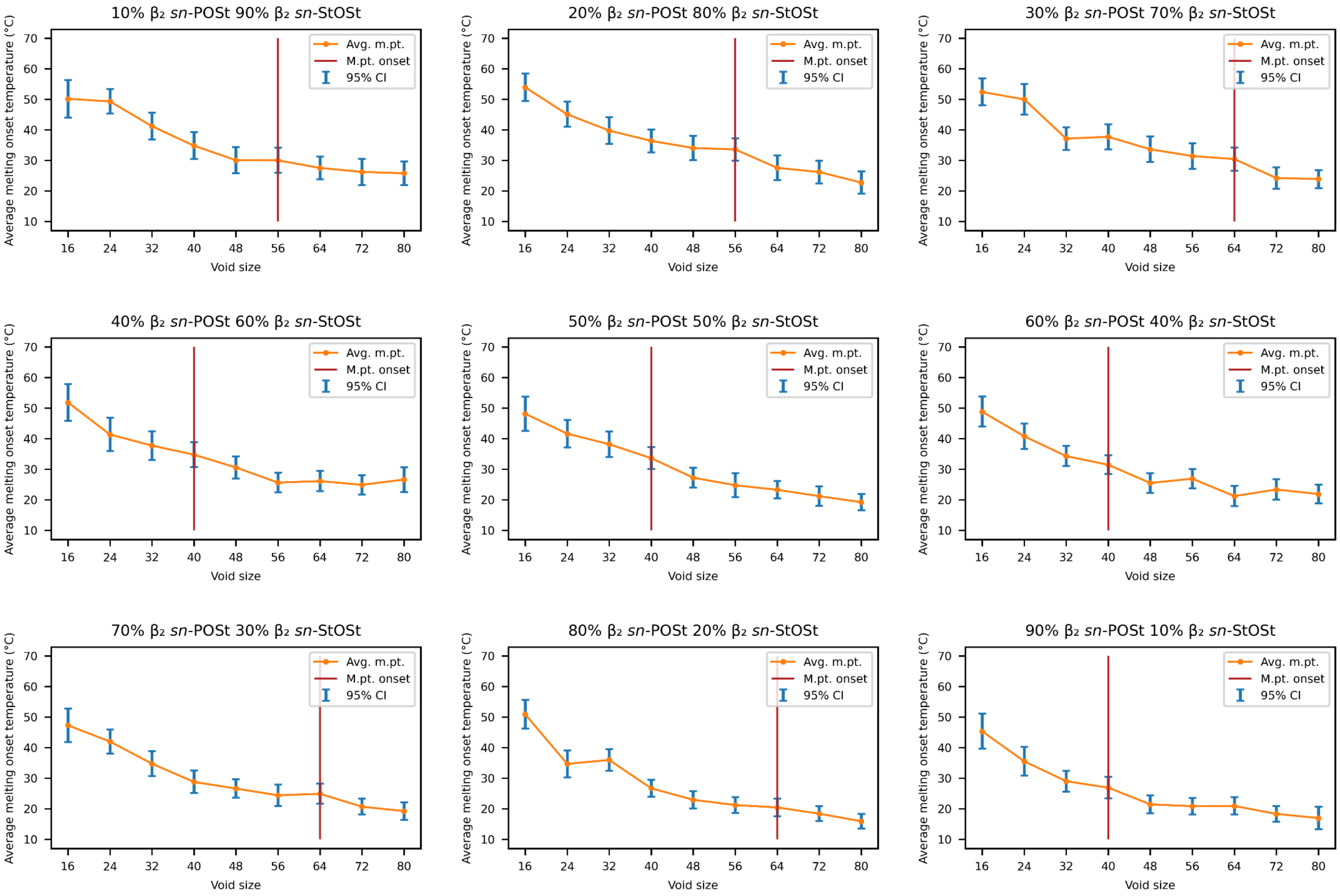

S.4 – Results for Different Void Sizes for Different *sn*-POP : *sn*-POSt ratios

*Table S.4a - Average Melting Onset Temperature*

| | % *sn*-POP | | | | | | | | |
|---|---|---|---|---|---|---|---|---|---|
| void size (molecules) | 10% | 20% | 30% | 40% | 50% | 60% | 70% | 80% | 90% |
| 16 | 41.77 | 45.15 | 41.18 | 39.18 | 40.59 | 38.96 | 38.99 | 37.48 | 39.65 |
| 24 | 32.70 | 31.67 | 32.05 | 29.84 | 30.79 | 32.43 | 30.19 | 31.95 | 32.09 |
| 32 | 28.54 | 26.39 | 25.34 | 24.86 | 24.35 | 27.24 | 24.51 | 27.23 | 28.31 |
| 40 | 20.54 | 21.29 | 21.78 | 22.39 | 21.58 | 22.56 | 23.15 | 24.37 | 23.46 |
| 48 | 18.67 | 18.94 | 20.45 | 18.98 | 19.42 | 21.12 | 19.23 | 18.58 | 21.63 |
| 56 | 19.07 | 17.63 | 15.55 | 14.41 | 18.75 | 17.44 | 17.40 | 17.56 | 16.49 |
| 64 | 17.07 | 15.81 | 16.50 | 16.09 | 14.75 | 15.64 | 16.34 | 14.23 | 16.82 |
| 72 | 18.44 | 13.03 | 15.93 | 13.43 | 15.65 | 12.72 | 14.23 | 13.68 | 14.40 |
| 80 | 17.47 | 17.29 | 16.87 | 13.96 | 13.79 | 13.97 | 13.90 | 14.96 | 12.84 |

*Table S.4b - 95% Confidence Interval*

| | % *sn*-POP | | | | | | | | |
|---|---|---|---|---|---|---|---|---|---|
| void size (molecules) | 10% | 20% | 30% | 40% | 50% | 60% | 70% | 80% | 90% |
| 16 | 5.16 | 3.32 | 3.75 | 4.42 | 3.60 | 3.65 | 3.58 | 3.92 | 3.50 |
| 24 | 4.00 | 4.21 | 2.96 | 2.59 | 3.43 | 3.16 | 3.48 | 3.26 | 2.87 |
| 32 | 3.10 | 3.10 | 2.98 | 2.45 | 2.77 | 3.13 | 3.22 | 3.05 | 3.60 |
| 40 | 2.60 | 2.48 | 2.48 | 2.45 | 2.88 | 2.88 | 3.08 | 3.00 | 2.92 |
| 48 | 2.43 | 2.41 | 2.38 | 2.24 | 2.98 | 3.18 | 2.51 | 2.72 | 3.29 |
| 56 | 2.20 | 2.66 | 2.08 | 2.03 | 3.23 | 2.76 | 3.01 | 2.69 | 2.52 |
| 64 | 2.48 | 2.64 | 2.29 | 2.17 | 2.60 | 2.22 | 2.79 | 2.29 | 3.00 |
| 72 | 4.07 | 2.54 | 2.53 | 2.34 | 2.80 | 1.80 | 2.20 | 3.42 | 2.67 |
| 80 | 2.87 | 4.48 | 3.56 | 2.34 | 3.23 | 1.79 | 3.11 | 4.19 | 2.41 |

*Figure S.4a-4i Plots of Average Melting Onset Temperature vs Void Size for mixtures of $\beta_2$ sn-POP and sn-POSt*

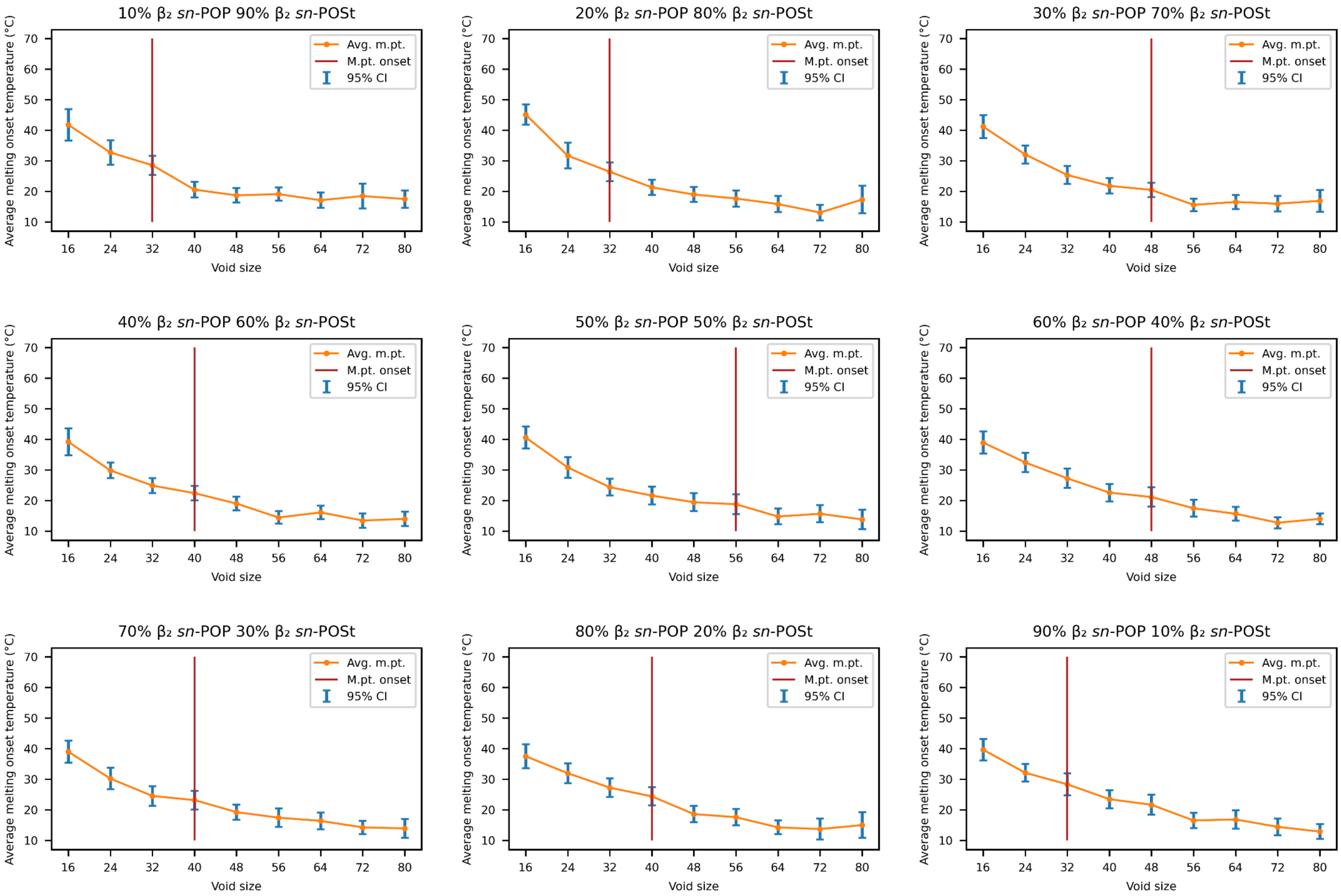

## S.5 – Results for Different Void Sizes for Different *sn*-POP : *sn*-StOSt ratios

*Table S.5a - Average Melting Onset Temperature*

| | % *sn*-POP | | | | | | | | |
|---|---|---|---|---|---|---|---|---|---|
| void size (molecules) | 10% | 20% | 30% | 40% | 50% | 60% | 70% | 80% | 90% |
| 16 | 55.18 | 50.52 | 50.97 | 45.60 | 42.16 | 40.87 | 38.52 | 36.05 | 33.88 |
| 24 | 46.01 | 43.90 | 43.94 | 38.58 | 33.70 | 32.82 | 30.63 | 28.62 | 29.80 |
| 32 | 43.51 | 39.48 | 32.88 | 32.88 | 30.28 | 27.80 | 25.21 | 24.53 | 25.77 |
| 40 | 36.10 | 36.61 | 30.31 | 28.94 | 28.75 | 25.04 | 22.02 | 20.59 | 21.12 |
| 48 | 34.29 | 33.81 | 30.39 | 26.71 | 21.12 | 23.56 | 19.49 | 16.86 | 18.69 |
| 56 | 32.98 | 29.73 | 26.21 | 23.75 | 22.08 | 19.03 | 15.70 | 13.56 | 16.32 |
| 64 | 29.37 | 23.63 | 23.92 | 21.04 | 18.09 | 16.20 | 14.63 | 15.60 | 13.42 |
| 72 | 26.86 | 25.15 | 25.05 | 20.06 | 16.15 | 17.04 | 14.55 | 13.01 | 13.12 |
| 80 | 23.51 | 23.95 | 20.93 | 19.75 | 18.64 | 16.43 | 16.44 | 11.17 | 14.82 |

*Table S.5b - 95% Confidence Interval*

| | % *sn*-POP | | | | | | | | |
|---|---|---|---|---|---|---|---|---|---|
| void size (molecules) | 10% | 20% | 30% | 40% | 50% | 60% | 70% | 80% | 90% |
| 16 | 5.41 | 5.01 | 4.43 | 4.87 | 4.43 | 4.70 | 3.32 | 3.73 | 3.70 |
| 24 | 5.09 | 5.21 | 4.11 | 3.84 | 3.75 | 4.17 | 2.83 | 2.76 | 3.33 |
| 32 | 3.99 | 4.72 | 4.47 | 3.91 | 3.32 | 2.94 | 2.88 | 2.79 | 3.17 |
| 40 | 4.39 | 4.70 | 4.03 | 3.14 | 2.68 | 3.10 | 3.26 | 2.68 | 2.42 |
| 48 | 4.61 | 3.89 | 3.69 | 3.34 | 3.06 | 2.91 | 2.94 | 2.55 | 2.74 |
| 56 | 4.50 | 3.96 | 3.46 | 3.47 | 3.07 | 2.52 | 1.88 | 1.99 | 3.08 |
| 64 | 4.12 | 3.44 | 3.48 | 2.93 | 2.78 | 2.13 | 2.09 | 2.58 | 2.21 |
| 72 | 3.46 | 3.87 | 3.87 | 2.80 | 2.19 | 2.71 | 2.14 | 2.01 | 3.04 |
| 80 | 3.89 | 3.74 | 3.09 | 2.87 | 3.59 | 2.84 | 2.71 | 1.78 | 4.01 |

*Figure S.5a-5i  Plots of Average Melting Onset Temperature vs Void Size for mixtures of $\beta_2$ sn-POP and sn-StOSt*

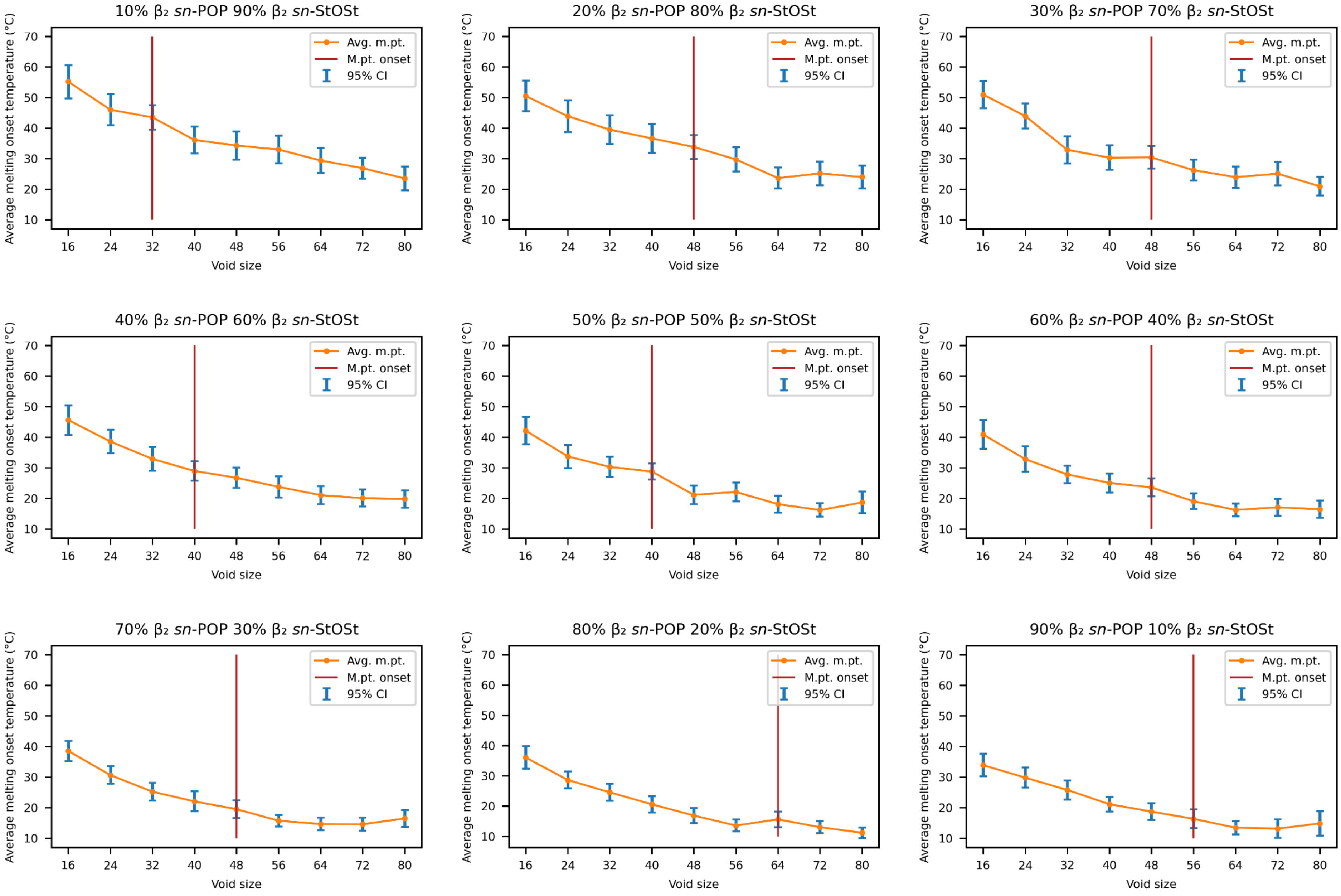

S.6 Plots of Average Melting Point Onset Temperature (°C) vs Void Size (determined using Volume change)

*Figure S.6a-6i Plots of Average Melting Onset Temperature vs Void Size for mixtures of $\beta_2$ sn-POP and sn-POSt*

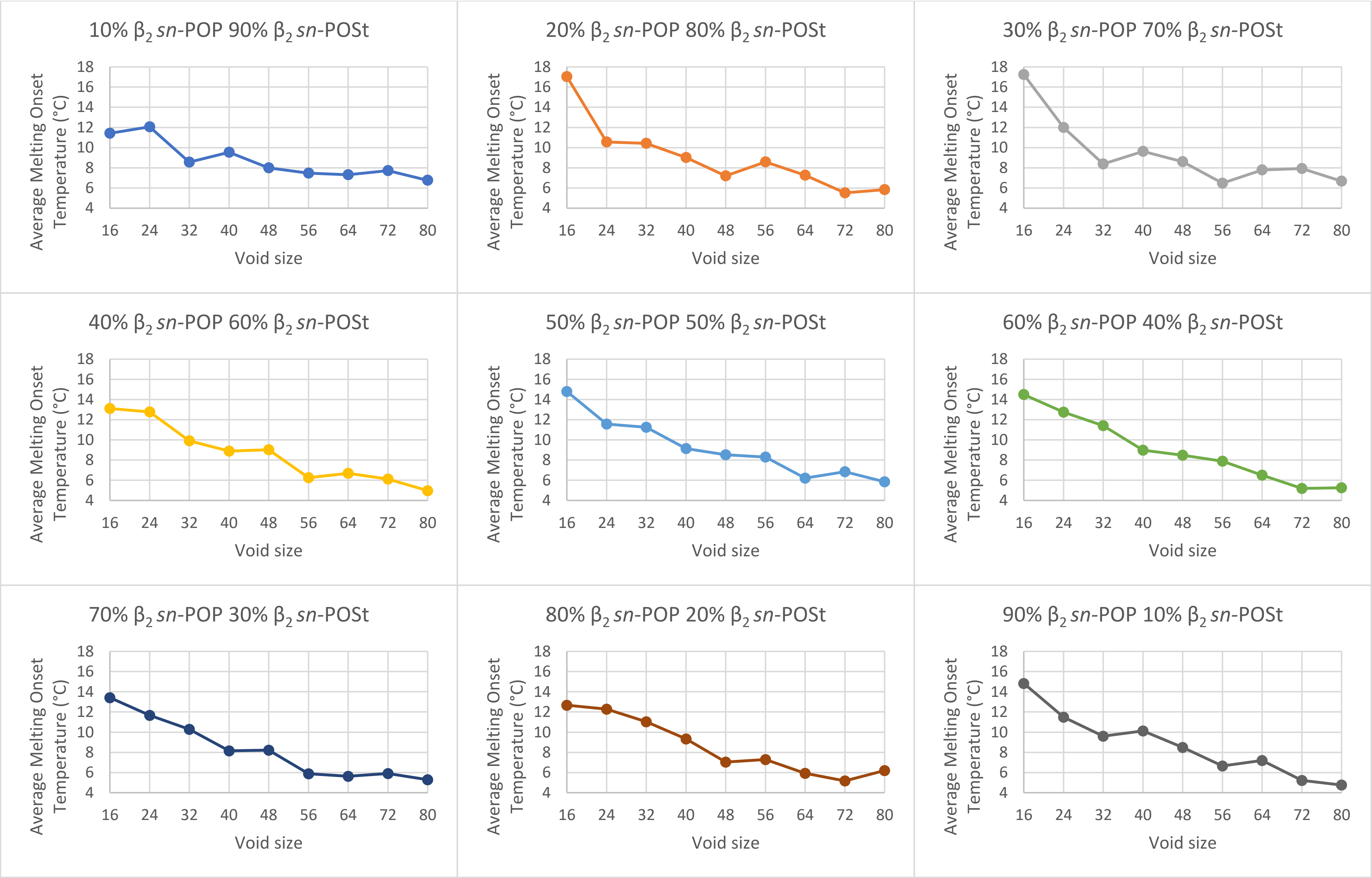

S.7 Plots of Average Melting Point Onset Temperature (°C) vs Void Size (determined using Volume change)

*Figure S.7a-7i Plots of Average Melting Onset Temperature vs Void Size for mixtures of $\beta_2$ sn-POSt and sn-StOSt*

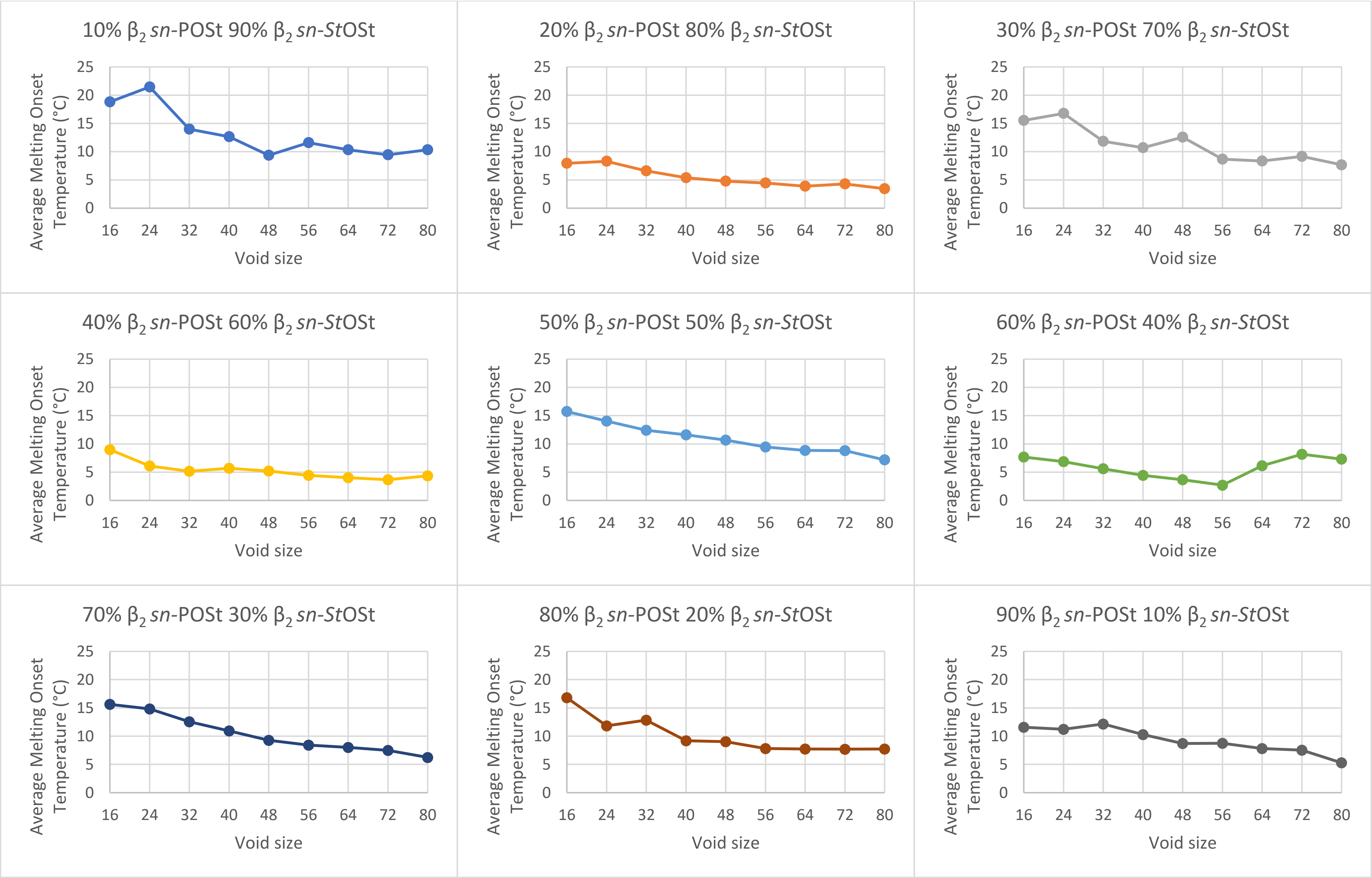

S.8 Plots of Average Melting Point Onset Temperature (°C) vs Void Size (determined using Volume change)

*Figure S.8a-8i  Plots of Average Melting Onset Temperature vs Void Size for mixtures of β₂ sn-POP and sn-StOSt*

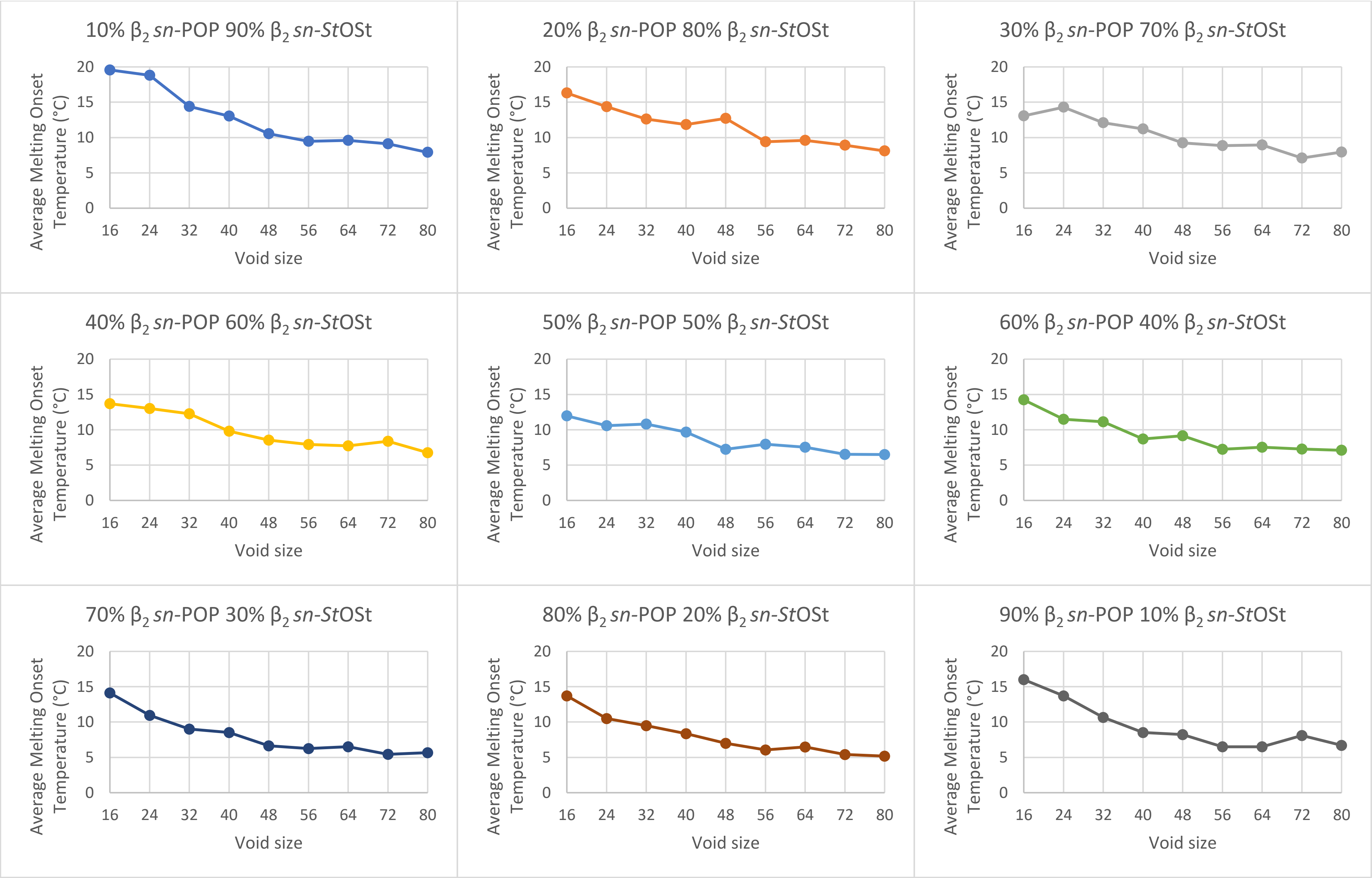

Supplement: Supplementary file 1 — jp3c06297_si_001.pdf [file jp3c06297_si_001.pdf]
